# Inter- and intra-tree variability of carbon and oxygen stable isotope ratios of modern pollen from nine European tree species

**DOI:** 10.1371/journal.pone.0234315

**Published:** 2020-06-09

**Authors:** Carolina Müller, Manja Hethke, Frank Riedel, Gerhard Helle

**Affiliations:** 1 Institute of Geological Sciences, Palaeontology, Freie Universität Berlin, Berlin, Germany; 2 Section 4.3 Climate Dynamics and Landscape Evolution, GFZ German Research Centre for Geoscience, Potsdam, Germany; Baylor University, UNITED STATES

## Abstract

Stable carbon and oxygen isotope ratios of raw pollen sampled from nine abundant tree species growing in natural habitats of central and northern Europe were investigated to understand the intra- and inter-specific variability of pollen-isotope values. All species yielded specific δ^13^C_*pollen*_ and δ^18^O_*pollen*_ values and patterns, which can be ascribed to their physiology and habitat preferences. Broad-leaved trees flowering early in the year before leaf proliferation (*Alnus glutinosa* and *Corylus avellana*) exhibited on average 2.6‰ lower δ^13^C_*pollen*_ and 3.1‰ lower δ^18^O_*pollen*_ values than broad-leaved and coniferous trees flowering during mid and late spring (*Acer pseudoplatanus*, *Betula pendula*, *Carpinus betulus*, *Fagus sylvatica*, *Picea abies*, *Pinus sylvestris* and *Quercus robur*). Mean species-specific δ^13^C_*pollen*_ values did not change markedly over time, whereas δ^18^O_*pollen*_ values of two consecutive years were often statistically distinct. An intra-annual analysis of *B*. *pendula* and *P*. *sylvestris* pollen revealed increasing δ^18^O_*pollen*_ values during the final weeks of pollen development. However, the δ^13^C_*pollen*_ values remained consistent throughout the pollen-maturation process. Detailed intra-individual analysis yielded circumferential and height-dependent variations within carbon and oxygen pollen-isotopes and the sampling position on a tree accounted for differences of up to 3.5‰ for δ^13^C_*pollen*_ and 2.1‰ for δ^18^O_*pollen*_. A comparison of isotope ranges from different geographic settings revealed gradients between maritime and continental as well as between high and low altitudinal study sites. The results of stepwise regression analysis demonstrated, that carbon and oxygen pollen-isotopes also reflect local non-climate environmental conditions. A detailed understanding of isotope patterns and ranges in modern pollen is necessary to enhance the accuracy of palaeoclimate investigations on δ^13^C and δ^18^O of fossil pollen. Furthermore, pollen-isotope values are species-specific and the analysis of species growing during different phenophases may be valuable for palaeoweather reconstructions of different seasons.

## Introduction

Stable carbon and oxygen isotope ratios of plant material are generally determined to understand the relationship between plants and their surrounding environment [1]. The continuously deepening knowledge of stable isotope patterns in plants of natural habitats finds applications in, for example, plant ecology, phytochemistry, genetic research and reconstructions of past environmental changes [2–6]. Plant physiological reactions to environmental factors, such as temperature, moisture availability and density of the surrounding vegetation, are known to affect the stable carbon and oxygen isotope composition of plant material [1, 7, 8]. Carbon isotope ratios (δ^13^C) of plant material are mostly determined by the factor-dependent amount of discrimination against ^13^C during CO_2_ uptake and by subsequent photosynthetic processes [e.g. 9, 10], while oxygen isotope ratios (δ^18^O) are often linked with the isotopic composition of environmental source water [11–13].

In general, the stable carbon isotope composition of stem material, leaves and pollen of the same plant individual highly correlate with one another [13–15]. Research on stable carbon isotope ratios of modern pollen focused mainly on species-specific patterns and ranges [15–18] and has been applied to investigate predominant photosynthetic pathways within grasslands [19–21]. Loader and Hemming [22] analysed δ^13^C_*pollen*_ of *Pinus sylvestris* from 28 sites across Europe and identified a positive linear correlation between δ^13^C values and the prevailing temperature during pollen formation. Also, Jahren [14] detected positive correlations of δ^13^C_*pollen*_ values with temperature for nine out of 14 plant species. Studies focussing on the determination of influencing climate factors on the isotope values of modern pollen include Bell et al. [15], who showed that δ^13^C_*pollen*_ of *Cedurs atlantica* (Atlas cedar) correlates with precipitation and a long-term annual and summer scPDSI (self-calibrating Palmer Drought Severity Index). Schwarz [13] suspected a relationship between δ^13^C_*pollen*_ and relative humidity, but no significant correlation of δ^13^C_*pollen*_ of *Pinus retinosa* (Red pine), *Pinus sylvestris* (Scots pine) and *Quercus rubra* (Northern red oak) could be detected at North American sampling sites. However, all correlations were highly species-dependent and several plants strongly reacted to other untested environmental factors, superimposing the climate signal archived in the pollen [14].

Little is known about the variability within modern oxygen pollen-isotope values. Nonetheless, they have already been successfully applied to determine the provenance of honey [e.g. 23, 24]. Loader and Hemming [25] identified a negative relationship of δ^18^O_*pollen*_ values with the δ^18^O values of precipitation during pollen formation, contrasting δ^18^O of wood and leaves that is typically positively related to the δ^18^O of precipitation [e.g. 26, 27]. Even if the δ^18^O_*pollen*_ is highly determined by the δ^18^O of local precipitation, the degree of dependence seems to vary with plant type and physiology [13]. Hence, δ^13^C_*pollen*_ and δ^18^O_*pollen*_ values are influenced by local climate conditions during pollen formation [15, 22], but not all variability within pollen-isotope values can be ascribed to climate-related environmental factors alone.

Morphology-based analysis of fossil pollen is frequently used to reconstruct palaeovegetation, since fossil pollen are often well preserved, widespread and abundant in various Cenozoic archives [28–34]. A combination of traditional pollen analysis and stable isotope analysis of fossil pollen might enhance environmental reconstructions in a high spatio-temporal resolution. Due to plant-specific timings in pollen production and pollen shedding, even intra-annual weather signals may be recorded in the δ^13^C_*pollen*_ and δ^18^O_*pollen*_ [13]. Some studies have already applied δ^13^C analysis to fossil pollen in an attempt to reconstruct past environmental changes [35–37]. A fossil δ^13^C_*pollen*_ record of *C*. *atlantica* from Morocco revealed the feasibility of reconstructing a long-term trend of increasing aridity by analysing species-specific pollen-isotopes [38]. Also, fossil *Nothofagus* (Southern beech) δ^13^C_*pollen*_ indicated different moisture availability in Antarctica during the early and middle Eocene [39].

However, interpretation of fossil pollen-isotopes are based on observations of modern δ^13^C_*pollen*_ and δ^18^O_*pollen*_ patterns and ranges. In addition to climate conditions during pollen formation, non-climate impact factors may need to be considered when interpreting the δ^13^C_*pollen*_ and δ^18^O_*pollen*_ values. These factors include site-specific environmental parameters such as type of soil, plant associations, position on slopes, and the proximity of the individual tree to perennial waterbodies. A comparison of several abundant tree species growing at different sites under the same environmental conditions, intra-tree differences and fluctuations over several vegetation periods helps to assess the impact of non-climate environmental factors on the pollen-isotope values.

In the present study, we address the species-specific natural variability of pollen-isotopes of nine abundant tree species across seven European sites ranging from Belgium to Poland and Finland to Italy, sampled during the years 2015 and 2016. The species have been chosen based on their widespread abundance in natural European forests and the frequency of their pollen in fossil records.

Inter- and intra-tree δ^13^C_*pollen*_ and δ^18^O_*pollen*_ isotope variabilities were assessed and tested for relationships with non-climate environmental factors by stepwise regression analysis. In doing so, we aimed to advance our understanding of pollen stable isotope signals for future palaeoclimate reconstructions. In detail we investigated:

1. Species-specific δ^13^C_*pollen*_ and δ^18^O_*pollen*_ patterns of selected tree taxa and the variability of their pollen-isotopes between two consecutive vegetation periods (2015/2016).
2. δ^13^C_*pollen*_ and δ^18^O_*pollen*_ at different stages of the pollen maturation process.
3. Variability of δ^13^C_*pollen*_ and δ^18^O_*pollen*_ at different heights and cardinal directions of individual trees.
4. δ^13^C_*pollen*_ and δ^18^O_*pollen*_ along a gradient of continentality (W–E transect) and along a gradient in day length (N–S transect).

## Material and methods

### Sampling locations, sample collection and preparation

**Fig 1:**
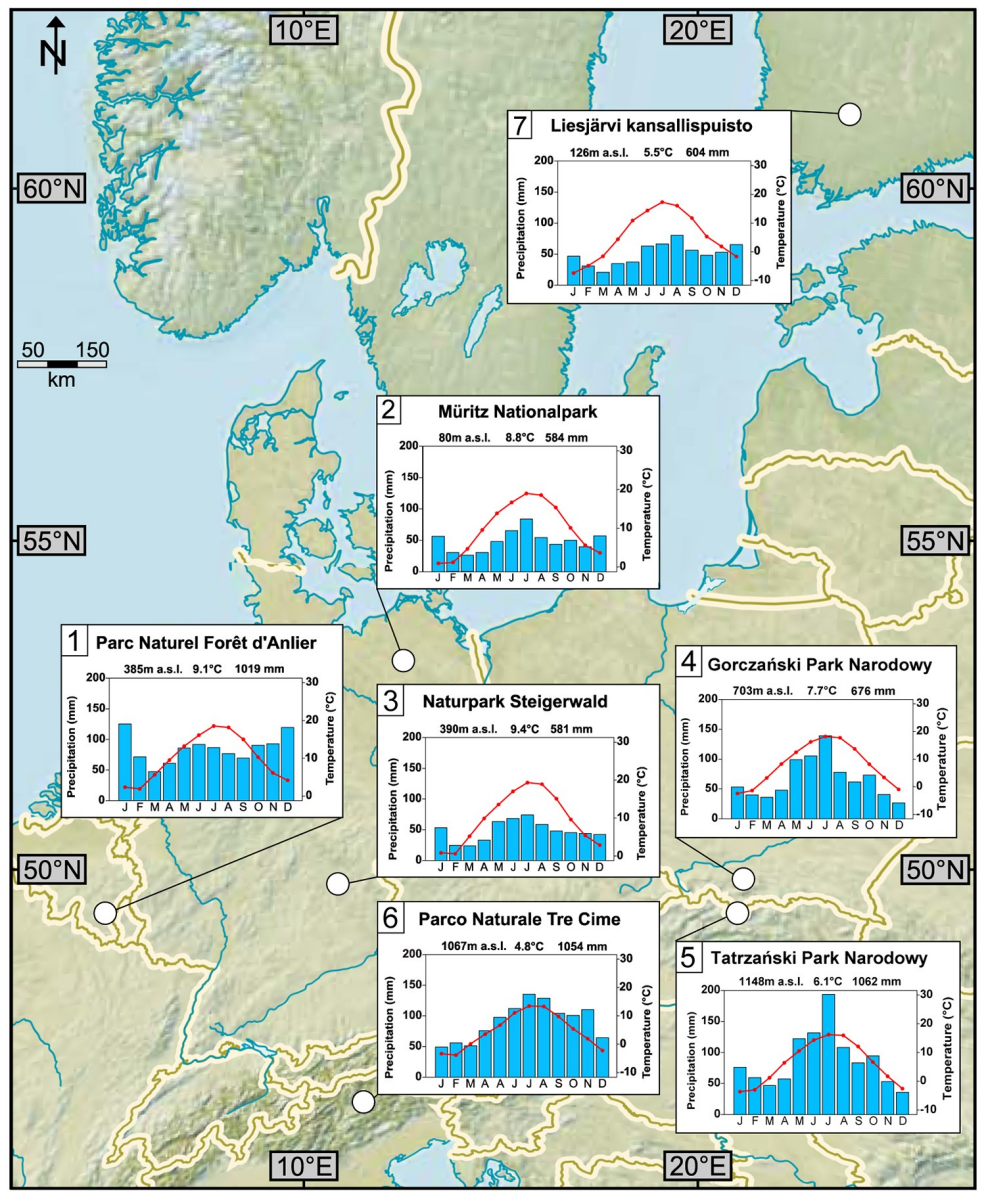
Study region (46.6°N– 53.3°N/5.7°E– 23.7°E). Topographic map of central Europe showing the sites (Nature Parks or National Parks) for pollen sampling (white dots) and respective climate diagrams with average seasonal temperature and precipitation. Refer to Table 1 for sampling site numbers and further details. Map modified from https://www.cia.gov/library/publications/the-world-factbook/index.html.

**Fig 2:**
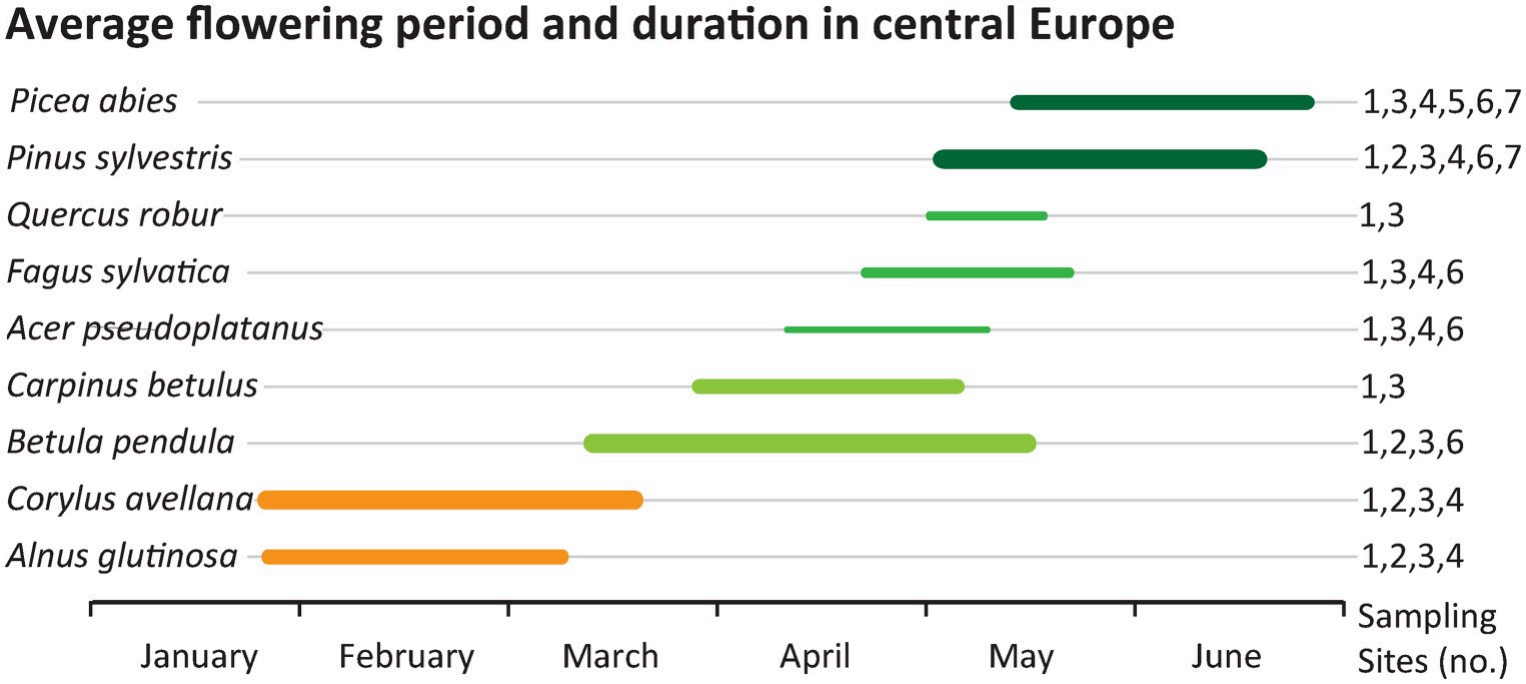
Average seasonal timing and duration of flowering periods in central Europe. Relative amount of pollen released by the nine examined species of this study indicated by line thickness in three steps (summarised from http://www.pollenstiftung.de and personal observation). Colours classify species according to their average blossoming time (orange: early blossoming, January to March; green colour saturation level indicates spring to early summer). The duration is an estimated average of species from central European locations. Sampling site (no.) indicates the sites where the various species have been sampled according to Table 1 and Fig 1.

We sampled modern pollen from 658 individual trees of nine selected tree species growing in seven national and nature parks, which we consider natural habitats (Fig 1; Tables 1 and 2). None of the species sampled in this study is endangered or protected and sampling followed generally a non-invasive scheme of few inflorescences per individual tree. Therefore, after contacting and consulting with the national park authorities of each sampling site, no specific permissions were required for these locations and activities. Pollen were collected during two consecutive vegetation periods (February to June of 2015 and 2016) within the species-specific flowering periods (Fig 2; Table 2). The schedule for sampling followed individual phenology and thus roughly the geographic distribution and climate conditions of west-east and south-north gradients in Europe [40]. All selected tree species use the C3 photosynthetic pathway. In principle, 20 flowering individuals were sampled per site and per species (Table 2). In case of dense forests, trees close to hiking trails, forestry roads or glades were sampled because sunlight illuminating the full height of tree crowns allows the development of lower branches with inflorescences. Samples were taken with a pruning device and an extendable stick from branches at positions of one metre up to seven metres above ground. Male inflorescences were cut off and placed in plastic bags. Bulk -samples of an individual tree were composed of several inflorescences of different branches from various heights. In the field, the samples were kept in a cooling box. Later, they were stored in a refrigerator at 6 °C to prevent mould infestation. In the laboratory, the samples were dried in a drying oven at a maximum temperature of 45 °C for seven to nine days. Dry samples were kept in a freezer at -16 °C until further processing. The separation of pollen from other flower tissue was achieved by thorough rinsing with deionised water using sieves with mesh sizes from 10 μm to 200 μm. Following rinsing and sieving the pollen were freeze-dried for 48–72 hours until fully dehydrated, transferred into Eppendorf (2 ml) safe lock tubes and frozen at -16 °C for preservation.

**Table 1:** Sampling locations and site characteristics.

Site	Site name	Park authority	Coordinates	Forest type and location characteristics	MAT (°C; min., max.),
No.	(country)	address	(altitude)	(soil type after WRB-FULL)	MAP
1	Parc naturel Forêt d’Anlier (Belgium)	Fédération des Parcs naturels de Wallonie Rue de Coppin, 20 5100 Jambes Tel. +32 81 30 21 81 e-mail: info@fpnw.be	49.7899° N 5.6829° E (385 m a.s.l.)	Mesophytic deciduous broad-leaved and mixed coniferous-broad-leaved forest; Beech and mixed beech forest, montane to altimontane type, partly with fir and spruce. (Dystric Cambisol)	9.1 (-0.1 to 16.1), 1019 mm
2	Müritz-Nationalpark (Germany)	Nationalparkamt Müritz Schlossplatz 3 17237 Hohenzieritz Tel. 039824/252-0 e-mail: poststelle@npa-mueritz.mvnet.de	53.3268° N 13.1925° E (80 m a.s.l.)	Mesophytic deciduous broad-leaved and mixed coniferous-broad-leaved forest; Beech and mixed beech forest, lowland to submontane type. (Haplic Luvisol)	8.8 (-0.9 to 17.2), 584 mm
3	Naturpark Steigerwald (Germany)	Naturpark Steigerwald e.V. Hauptstraße 1 91443 Scheinfeld Tel. 09161/92-1523 e-Mail: info@steigerwald-naturpark.de	49.8616° N 10.5241° E (390 m a.s.l.)	Mesophytic deciduous broad-leaved and mixed coniferous-broad-leaved forest; Mixed oak-hornbeam forest. (Dystric Cambisol)	9.4 (-1.2 to 17.5), 581 mm
4	Gorczański Park Narodowy (Poland)	Gorczański Park Narodowy Poręba Wielka 590 34–735 Niedźwiedź Tel. +48 33 17 207 e-mail: gpn@gorcepn.pl	49.5608° N 20.1614° E (703 m a.s.l.)	Mesophytic deciduous broad-leaved and mixed coniferous-broad-leaved forest; Beech and mixed beech forest, montane to altimontane type, partly with fir and spruce. (Haplic Leptosol/ Dystric Cambisol)	7.7 (-3.8 to 17.5), 676 mm
5	Tatrzański Park Narodowy (Poland)	Tatrzański Park Narodowy Kuźnice 1 34–500 Zakopane Tel. +48 18 20 23 200 e-mail: sekretariat@tpn.pl	49.2571° N 19.9691° E (1148 m a.s.l.)	Mesophytic and hygromesophytic coniferous and mixed broad-leaved-coniferous forest; Montane to altimontane, partly submontane fir and spruce forests in the nemoral zone. (Calcaric Leptosol/ Dystric Leptosol)	6.1 (-5.3 to 15.3), 1062 mm
6	Parco Naturale Tre Cime (Italy)	Amt für Natur Landhaus 11 Rittner Straße 4 39100 Bozen Tel. +39 0471 41 77 70 e-mail: natur.bozen@provinz.bz.it	46.6412° N 12.3374° E (1067 m a.s.l.)	Mesophytic deciduous broad-leaved and mixed coniferous-broad-leaved forest; Beech and mixed beech forest, montane to altimontane type, partly with fir and spruce. (Rendzic Leptosol)	4.8 (-5.8 to 15.0), 1054 mm
7	Liesjärvi kansallispuisto (Finland)	Metsähallitus P.O. Box 94 (Ratatie 11) FI-01301 Vantaa Tel. +358 206 39 4000	60.6633° N 23.8797° E (126 m a.s.l.)	Mesophytic and hygromesophytic coniferous and mixed broad-leaved-coniferous forest; Southern boreal type. (Haplic Podzol)	5.5 (-7.1 to 16), 604 mm

Sampling site overview including park authority address with contact details for sampling permissions, geographic coordinates, average elevation above sea level, forest and soil classification, average temperatures and mean annual precipitation. Forest classifications follow the “General Map of the Natural Vegetation of Europe” (Federal Agency for Nature Conservation, Bonn 2001). Soil classifications according to the European Soil Data Centre [41]; MAP (mean annual precipitation) and MAT (mean annual temperature; including the mean temperature of the coldest and warmest month) have been calculated based on the high-resolution gridded dataset CRU TS at http://www.cru.uea.ac.uk/data.

**Table 2:** Overview of species.

Taxonomy	Common name	Flowering period	Individuals	Individuals
			2015	2016
Coniferophyta				
Pinaceae				
*Picea abies* (L.) H. KARST.	Norway spruce	May/June	63	65
*Pinus sylvestris* (L.)	Scots pine	May/June	69	59
Magnoliopsida				
Sapindaceae				
*Acer pseudoplatanus* (L.)	Sycamore	April/May	9	24
Betulaceae				
*Alnus glutinosa* (L.) GAERTN.	Black alder	January/March	43	49
*Betula pendula* ROTH	Silver birch	April/May	33	38
*Carpinus betulus* (L.)	European hornbeam	April/May	1	18
*Corylus avellana* (L.)	Common hazel	January/March	43	49
Fagaceae				
*Fagus sylvatica* (L.)	European beech	May	11	46
*Quercus robur* (L.)	Pendunculate oak	May	16	22

Taxonomic classification of the nine investigated tree species including their common names and specific flowering periods. The number of individuals sampled represents the sum of trees sampled at all sites in 2015 and 2016, respectively.

**Fig 3:**
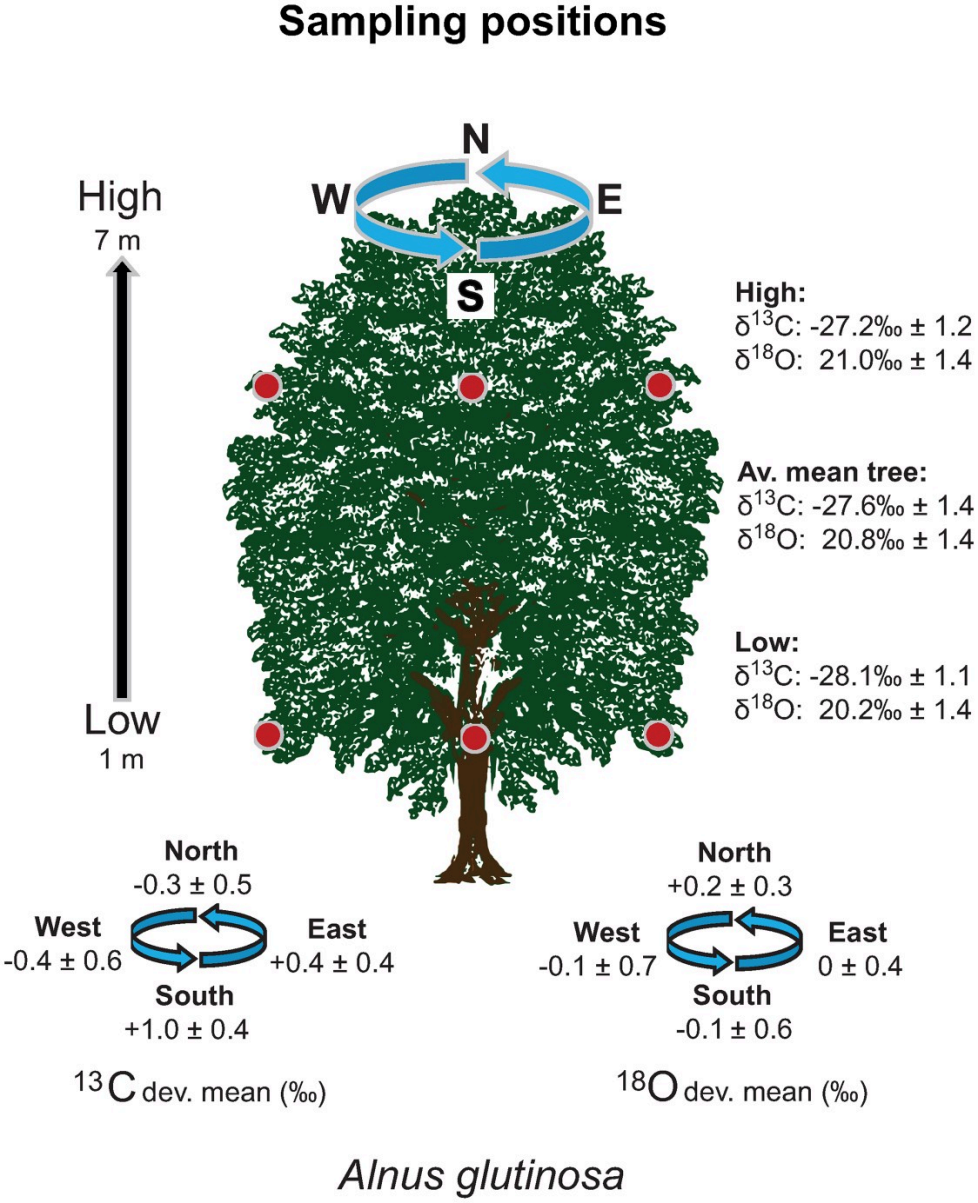
Intra-tree pollen-isotope variability of *Alnus glutinosa*. The sampling scheme for intra-tree pollen-isotope analysis comprises the sampling of pollen from each cardinal direction at a low and a high position in a tree (1 m and 7 m above ground; red dots). Average isotope values (Av. mean tree) as well as the values of both the high and low positions are given for one exemplary *Alnus glutinosa* tree. ^13^C and ^18^O dev. mean = average deviation from the mean isotope value of the tree.

To investigate intra-tree isotope patterns, an additional 152 intra-tree sub-samples were taken at different heights and cardinal directions from 22 trees of eight species (Fig 3). In addition, individual inflorescences were collected separately in plastic bags to allow a high-resolution intra-tree variance analysis.

Intra-annual analyses at different stages of the pollen maturation process were carried out for *B*. *pendula* and *P*. *sylvestris*, which were sampled twice at the same location within one vegetation period. *Betula pendula* was collected at Forêt d’Anlier on 10 March and 5 May 2015, whereas *P*. *sylvestris* was sampled twice in Gorczański National Park on the 20 May and 1 June 2015. The individual trees sampled for the intra-annual analysis grew within a small assessable area with similar habitat conditions. Due to minimal individual offsets in flower development and senescence, only some tree individuals could be sampled and analysed twice. Seven of the *P*. *sylvestris* individuals were identical (total number of samples at first sampling: 12; total number of samples at second sampling: 16) and eight *B*. *pendula* trees were samples twice (total number of samples at first sampling: 11; total number of samples at second sampling: 22). An elevation transect in the Tatrzański Mountains National Park extends from 1053 m a.s.l. to 1345 m a.s.l. on a north-facing slope. Along the gradient of roughly 300 m, 15 individual trees of *Picea abies* were sampled at five different elevations in 2015 and 2016.

### Stable isotope analysis

For each measurement, the amount of 220 μg ± 10% of chemically untreated pollen material was weighed directly into silver capsules using a high-precision scale (Mettler Toledo AX 26 Delta Range). δ^13^C and δ^18^O were determined using a DELTA V isotope ratio mass spectrometer (IRMS; Thermo Fisher Scientific^™^, Bremen) at the dendrochronological laboratory, section 4.3, GFZ Potsdam, Germany. To exclude potential water contamination from air humidity, all samples were vacuum dried at 100 °C for at least 12 hours in a Thermo Scientific Heraeus VT 6060 P prior to measurement. The pollen material was reduced to CO for simultaneous IRMS analysis of carbon and oxygen isotope ratios in a High Temperature Conversion Elemental Analyzer (TC/EA; 1400 °C; Thermo Fisher Scientific^™^, Bremen) coupled to the IRMS. All isotope ratios are expressed relative to VPDB for δ^13^C and VSMOW for δ^18^O. Isotope data were compared against international and lab-internal reference material (IAEA-CH3, IAEA-CH6 and IAEA 601 and 602) using two reference standards with widespread isotopic compositions for a single-point normalisation [42]. Most of the 809 individual pollen samples were weighed and measured with two or three repetitions. In total, we conducted 2132 measurements of stable isotopes. The pollen-isotope dataset is deposited at Pangea Database (https://doi.org/10.1594/PANGAEA.910977).

### Statistical analysis

All calculations and graphics were done using programmes R [43] and RStudio [44]. Value distributions for each site and year with indicated average are shown as bean plots [45]. Many parametric tests assume a normal distribution. Hence, we tested whether the pollen-isotope values in a given sample of a species from each location and year were normally distributed using the Shapiro-Wilk test. The minimum sample size analysed was three. The null hypothesis of normal distribution was rejected, if the probability *p* was smaller than the significance level (range: 0–1; significance level: *p* = 0.05). As several distributions were non-normal, we used the non-parametric Mann-Whitney U test for equality of medians (*p(sm)*) to compare inter-annual distribution patterns of pollen-isotopes (range: 0–1; significance level: *p(sm)* = 0.05). A possible relationship between isotope values and elevation at Tatrańzki Park Narodowy (Poland) was investigated using linear correlation. Inter- and intra-tree variability of sub-samples taken at different tree heights and cardinal directions was characterised by calculating respective standard deviations (1σ).

### Stepwise regression analysis

Variables influencing the stable isotope composition in pollen (δ^13^C, δ^18^O) were explored by means of stepwise regression as implemented by the JMP Pro 13.1.0 software. Stepwise regression reduces variance to a linear model by eliminating insignificant predictors and was performed (1) by species (to investigate the most important influencing factors for each species over several sites) and (2) by site (to evaluate possible location-dependent environmental factors for the pollen-isotopes). For the analysis by species eight potential predictors entered our models, including the categorical variables *year* (of sampling: 2015 and 2016), *month* (of sampling: February to June), *maturity* (of the pollen at the time of sampling: -1 = immature, 0 = mature, 1 = withered inflorescence), *slope* (steepness: 0 = flat, 1 = minimal incline, up to 10°, 2 = moderate incline, more than 10°), *water* (proximity to water body: 0 = none in the direct vicinity, 1 = one between 10 and 20 m away, 2 = one up to ten metres away), *water classification* (type of water body, e.g. river, lake, wetland), *soil* (type of soil; Table 1) and the predictor *site* as a site-specific combination of latitude, longitude and altitude. For the second analysis (by site) the continuous variable *altitude* was additionally included to the categorical variables *year*, *month*, *maturity*, *slope*, *water*, *water classification*, *soil* and *species* and thus, nine potential predictors entered the second model. The factors were noted for each individual tree during field work. For all analyses, we deleted singletons by having a look at column variation (< 5 values in a column), hence, several analyses were performed using a subset of the predictors mentioned above.

Categorical variables were hierarchically coded by maximizing the sum of squares between groups. Therefore, the analysis also informs about how levels in categorical predictors are associated with each other. For example, a notation such as *site*{FAN&STE&GOR&LIE-TAT&TRE} (SI Dataset 1 and 2) contrasts sites Forêt d’Anlier, Steigerwald, Gorczański, and Liesjärvi against Tatrzański and Tre Cime.

Variables revealing the highest statistical significance were added to the model in a stepwise process. As a stopping rule for adding terms we used the minimum Bayesian Information Criterion. Subsequently, the model parameters were estimated using least squares regression.

## Results

δ^13^C_*pollen*_ and δ^18^O_*pollen*_ of 809 samples of 658 individual trees from nine common tree species have been analysed (Table 3). The samples were taken at seven locations across Europe during three different time periods of flowering (January to March, April to May and May to June).

**Table 3:** δ^13^C and δ^18^O of studied pollen.

Site name (no.)	Species	Time of flowering	Year	Samples (no.)	δ^13^C	δ^13^C	δ^13^C	δ^13^C	δ^18^O	δ^18^O	δ^18^O	δ^18^O
					mean	min.	max.	sd	mean	min.	max.	sd
Parc Naturel Forêt d’Anlier (1)	*Acer pseudoplatanus*	B	2016	6	-25.2	-27.1	-22.5	1.7	25.7	25.0	26.7	0.6
	*Alnus glutinosa*	A	2015	25	-28.6	-30.9	-25.9	1.5	22.0	21.0	23.1	0.6
		A	2016	33	-29.3	-32.3	-25.5	2.0	22.0	21.1	22.6	0.4
	*Betula pendula*	B	2015/1	11	-25.6	-26.9	-23.9	0.9	20.9	19.9	21.4	0.7
		B	2015/2	22	-25.7	-28.5	-23.3	1.5	24.9	22.2	26.5	1.2
		B	2016	15	-24.1	-25.8	-21.4	1.1	24.2	22.5	25.6	0.9
	*Carpinus betulus*	B	2015	1	-25.2	NA	NA	NA	29.0	NA	NA	NA
		B	2016	8	-26.1	-29.3	-23.4	2.2	26.3	25.3	27.5	0.6
	*Corylus avellana*	A	2015	27	-29.0	-30.7	-27.6	0.8	22.8	22.1	23.4	0.4
		A	2016	30	-28.6	-32.2	-26.3	1.5	22.0	20.7	23.6	0.7
	*Fagus sylvatica*	B	2016	23	-27.3	-29.3	-24.7	1.6	23.2	22.4	24.3	0.5
	*Picea abies*	C	2015	15	-26.2	-29.0	-23.5	1.3	24.0	23.1	25.4	0.8
		C	2016	22	-25.8	-28.0	-23.9	1.4	25.1	23.7	26.2	0.7
	*Pinus sylvestris*	C	2015	12	-27.8	-29.4	-26.3	1.0	25.2	21.1	28.7	2.7
		C	2016	20	-25.7	-27.8	-23.9	1.2	29.2	26.8	30.8	1.2
	*Quercus robur*	B	2015	16	-26.4	-29.0	-25.6	0.9	26.2	24.0	29.0	1.4
		B	2016	21	-25.7	-27.4	-22.8	1.5	27.4	25.1	29.2	1.1
Parco Naturale Tre Cime (6)	*Acer pseudoplatanus*	B	2015	3	-25.5	-27.3	-24.6	1.6	24.7	23.9	25.1	0.7
		B	2016	4	-26.1	-26.7	-25.0	0.8	23.1	21.8	24.3	1.0
	*Betula pendula*	B	2016	4	-24.8	-26.1	-23.8	1.0	23.9	22.9	25.2	1.0
	*Fagus sylvatica*	B	2016	4	-27.6	-28.1	-27.2	0.4	24.1	23.1	26.1	1.4
	*Picea abies*	C	2015	9	-23.8	-25.7	-22.3	1.1	23.5	21.0	26.0	1.5
		C	2016	31	-23.2	-24.6	-22.0	0.9	21.6	20.0	23.2	0.9
	*Pinus sylvestris*	C	2015	20	-26.3	-27.7	-25.1	0.7	26.0	24.4	26.9	0.8
		C	2016	14	-27.0	-29.1	-25.3	1.1	23.6	21.2	26.0	1.9
Gorczański Park Narodowy (4)	*Acer pseudoplatanus*	B	2015	6	-25.5	-28.3	-23.2	1.7	18.0	16.2	19.7	1.1
		B	2016	11	-24.8	-27.9	-23.3	1.4	18.5	16.9	20.2	1.0
	*Alnus glutinosa*	A	2015	26	-27.4	-29.6	-25.2	1.4	18.1	16.9	19.2	0.6
		A	2016	26	-26.4	-29.9	-22.3	1.9	19.1	17.6	21.7	1.1
	*Corylus avellana*	A	2015	29	-27.8	-30.4	-24.6	1.2	18.5	16.0	21.9	1.4
		A	2016	27	-26.5	-30.4	-24.4	1.6	19.2	17.5	21.2	1.3
	*Fagus sylvatica*	B	2015	10	-27.9	-28.9	-26.4	0.8	18.6	17.4	20.5	0.9
		B	2016	13	-27.4	-29.5	-24.9	1.8	19.5	17.4	22.1	1.3
	*Picea abies*	C	2015	17	-25.6	-27.8	-23.2	1.23	24.8	22.5	26.5	1.0
		C	2016	26	-25.8	-26.8	-24.1	0.8	21.5	20.5	22.4	0.6
	*Pinus sylvestris*	C	2015/1	12	-26.3	-27.5	-25.2	0.7	25.7	24.2	26.7	0.8
		C	2015/2	52	-26.3	-27.9	-23.9	1.3	26.9	25.6	28.4	0.8
		C	2016	13	-26.9	-28.9	-25.6	1.1	25.0	22.0	27.4	1.4
Liesjärvi	*Picea abies*	C	2016	10	-25.6	-27.4	-24.3	0.7	22.7	21.7	24.0	0.7
kan. (7)	*Pinus sylvestris*	C	2016	20	-27.9	-29.6	-26.5	0.8	23.6	21.6	24.6	0.7
Müritz NP (2)	*Alnus glutinosa*	A	2016	5	-31.0	-32.3	-30.0	1.0	22.5	22.2	23.2	0.4
	*Betula pendula*	B	2016	13	-23.9	-26.4	-22.1	1.7	24.1	25.2	25.1	0.6
	*Corylus avellana*	A	2016	6	-25.3	-27.6	-23.0	1.7	23.9	23.7	24.1	0.1
	*Pinus sylvestris*	C	2015	6	-26.9	-28.8	-26.2	0.9	28.8	28.1	29.4	0.5
Steigerwald National Park (3)	*Acer pseudoplatanus*	B	2016	9	-23.7	-24.8	-23.0	1.0	24.6	24.0	25.4	0.7
	*Alnus glutinosa*	A	2016	2	-26.6	-28.1	-25.2	2.1	22.9	22.9	22.9	0.0
	*Carpinus betulus*	B	2016	10	-25.6	-27.1	-23.5	1.4	26.1	24.7	27.3	0.9
	*Corylus avellana*	A	2016	2	-27.0	-27.6	-26.4	0.8	23.8	23.7	23.9	0.1
	*Betula pendula*	B	2016	9	-22.9	-25.0	-21.1	1.3	24.8	24.0	25.6	0.6
	*Fagus sylvatica*	B	2016	10	-25.5	-27.1	-23.6	1.1	23.4	22.4	24.9	0.8
	*Picea abies*	C	2015	7	-26.1	-28.8	-24.9	1.3	25.2	22.6	26.9	1.6
		C	2016	5	-25.8	-27.4	-23.6	1.5	21.7	19.6	22.9	1.3
	*Pinus sylvestris*	C	2015	5	-26.0	-27.3	-23.9	1.4	27.6	26.3	28.7	1.0
	*Quercus robur*	B	2016	3	-23.3	-23.6	-22.8	0.4	26.3	26.2	26.5	0.1
Tatrzański	*Picea abies*	C	2015	18	-24.3	-26.4	-22.1	1.1	22.6	21.1	23.9	0.8
PN (5)		C	2016	5	-26.3	-26.6	-26.0	0.3	21.3	21.0	21.9	0.5

Stable isotope values of δ^13^C_pollen_ and δ^18^O_pollen_ (min., max., standard deviation) differentiated by sample location (site name and assigned number), species and year. Flowering periods are indicated by letters: (A) January to March, (B) April to May and (C) May to June. The number of samples includes bulk samples of individual trees and sub-samples of different positions within a tree. Missing data is denoted as NA.

### δ^13^C_*pollen*_ values of broad-leaved and coniferous tree species

#### Flowering period January to March: *Alnus glutinosa* and *Corylus avellana*

**Fig 4:**
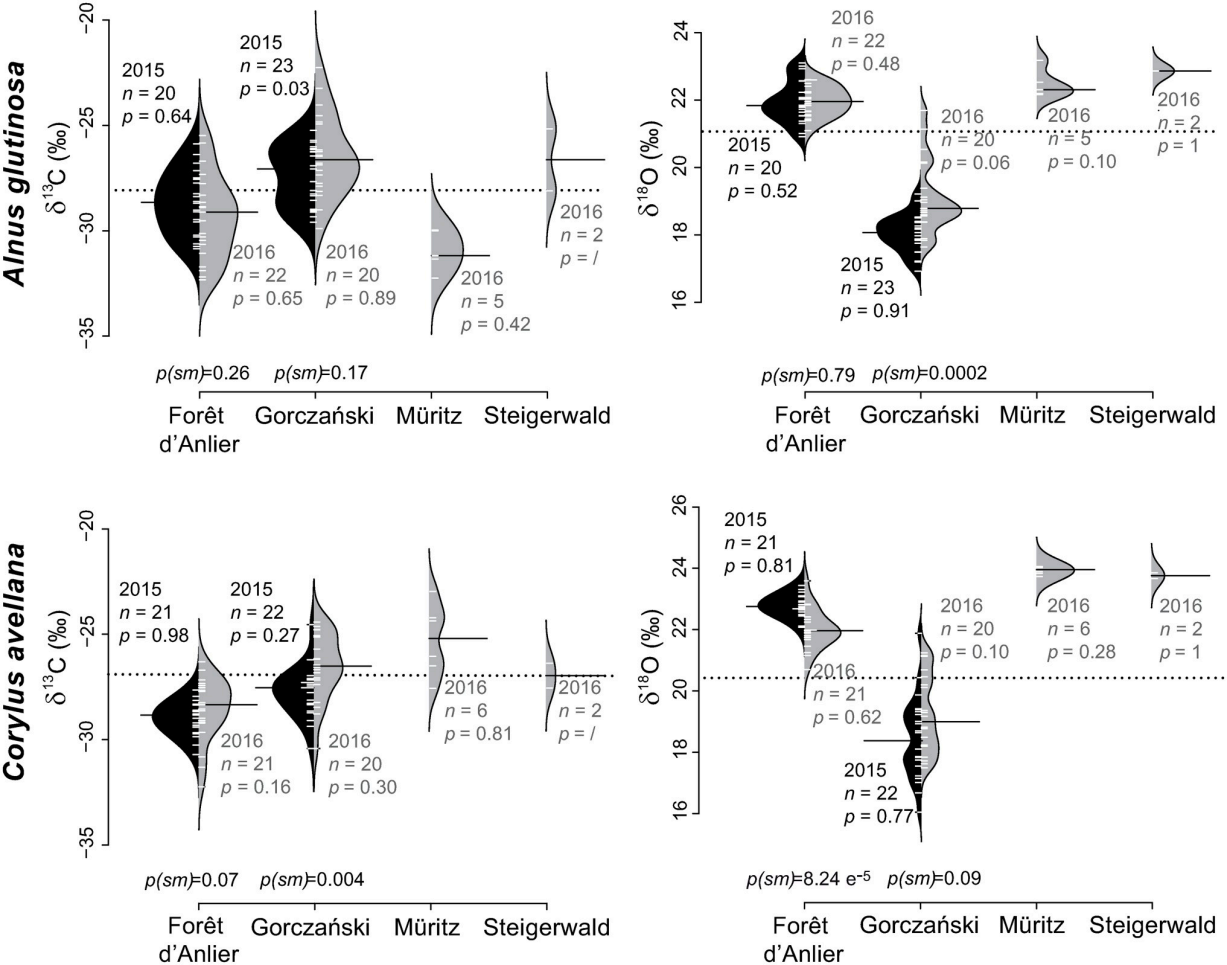
Pollen-isotopes of broad-leaved species flowering January to March: *Alnus glutinosa* and *Corylus avellana*. The bean plots show the values of δ^13^C_*pollen*_ and δ^18^O_*pollen*_ of the two species, *Alnus glutinosa* and *Corylus avellana*, from four different locations (Fig 1) sampled in 2015 (black) and 2016 (grey). Localities Steigerwald and Müritz were only sampled in 2016. *n* indicates the number of trees sampled on each occasion (i.e. year). *p-*values indicate whether the pollen-isotope values of one year are normally distributed, whereas *p(sm)* represents the probability for equal medians in samples of two consecutive years. The dotted line represents the mean over all localities and both years. The means of each sampling are indicated by a black bar.

The range of mean δ^13^C_*pollen*_ values from *A*. *glutinosa* for all sites and both years is 4.6‰ (-31.0‰ to -26.4‰; Table 3). With the exception of the Gorczański site (2015) the δ^13^C_*pollen*_ values of two consecutive years yield comparable medians at *p(sm)* = 0.26 and 0.17 (Fig 4). Mean δ^13^C_*pollen*_ values of *C*. *avellana* pollen range from -29.1‰ to -25.3‰ (3.8‰; Table 3) and are normally distributed. Similar medians for both years are suggested for Forêt d’Anlier at *p(sm)* = 0.07, while the null hypothesis of equal medians was rejected at Gorczański at *p(sm)* = 0.004.

#### Flowering period April to May: *Acer pseudoplatanus*, *Betula pendula*, *Carpinus betulus*, *Fagus sylvatica* and *Quercus robur*

**Fig 5:**
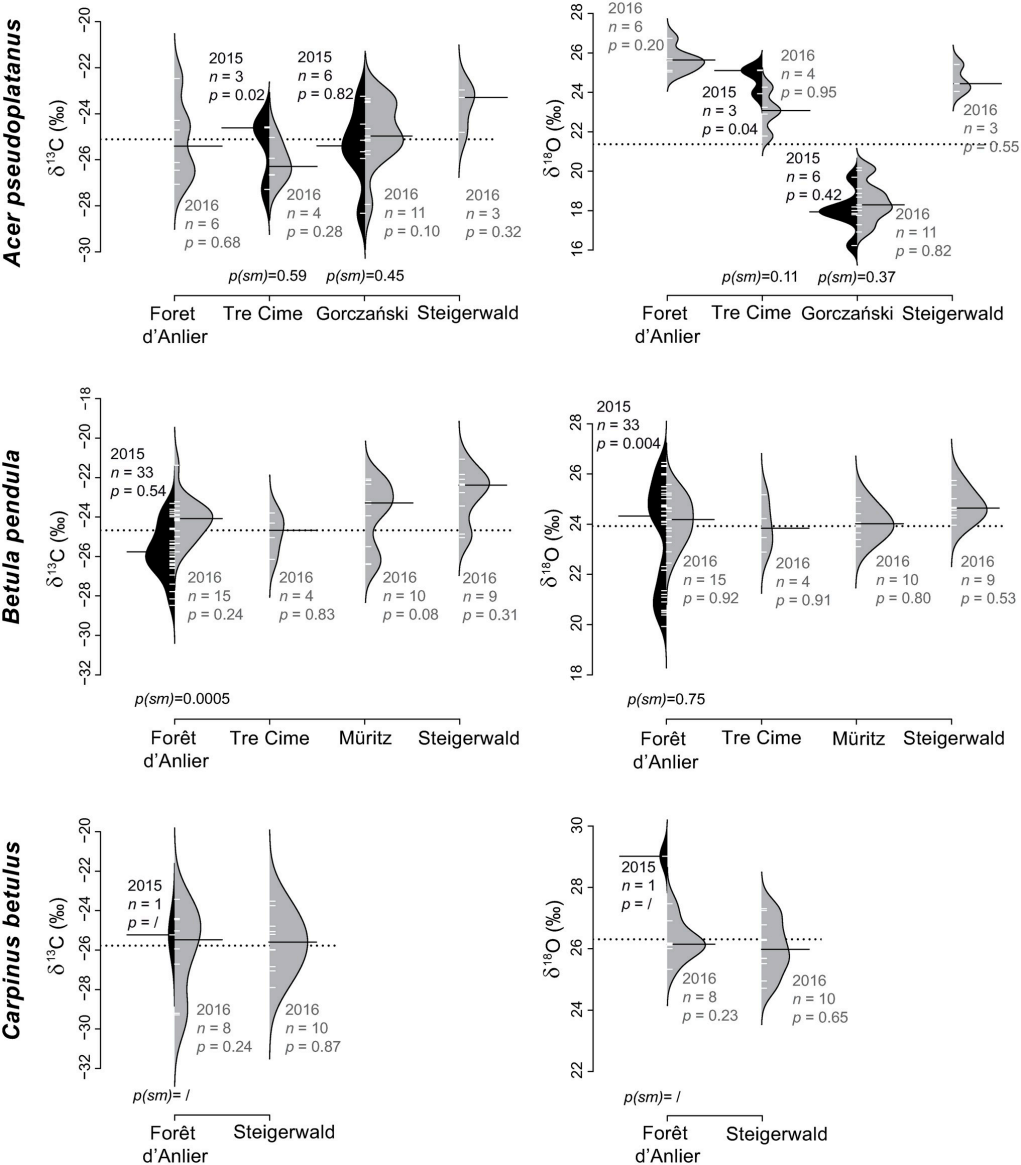
Pollen-isotopes of broad-leaved species flowering April to May: *Acer pseudoplatanus*, *Betula pendula* and *Carpinus betulus*. The broad-leaved species *Acer pseudoplatanus*, *Betula pendula* and *Carpinus betulus* were sampled at two to four locations. The bean plots show δ^13^C_*pollen*_ values (left) and δ^18^O_*pollen*_ values (right) of 2015 (black) and 2016 (grey). *n* indicates the number of individuals. *p-*values indicate whether pollen-isotope values of a single year are normally distributed (sign. level = 0.05), whereas *p(sm)* represents the probability for equal medians in samples of two consecutive years. The dotted line represents the mean over all localities and both years. The means of each sampling are indicated by a black bar.

Mean δ^13^C_*pollen*_ values of *A*. *pseudoplatanus* range from -26.1‰ to -23.7‰ (2.4‰; Table 3, Fig 5). With one exception at Tre Cime (2015, *p* = 0.02), the δ^13^C_*pollen*_ values of all sites are normally distributed and they yield similar medians for both vegetation periods. δ^13^C_*pollen*_ values of *B*. *pendula* are also normally distributed. Their mean ranges from -25.7‰ to -22.9‰ (2.8‰; Table 3, Fig 5), but the distributions yield unequal medians for both years at the site Forêt d’Anlier (*p(sm)* = 0.0005). Mean δ^13^C_*pollen*_ of *C*. *betulus* range between -26.1‰ and -25.6‰ (0.5‰) and the values reveal a normal distribution at each site (Table 3, Fig 5).

**Fig 6:**
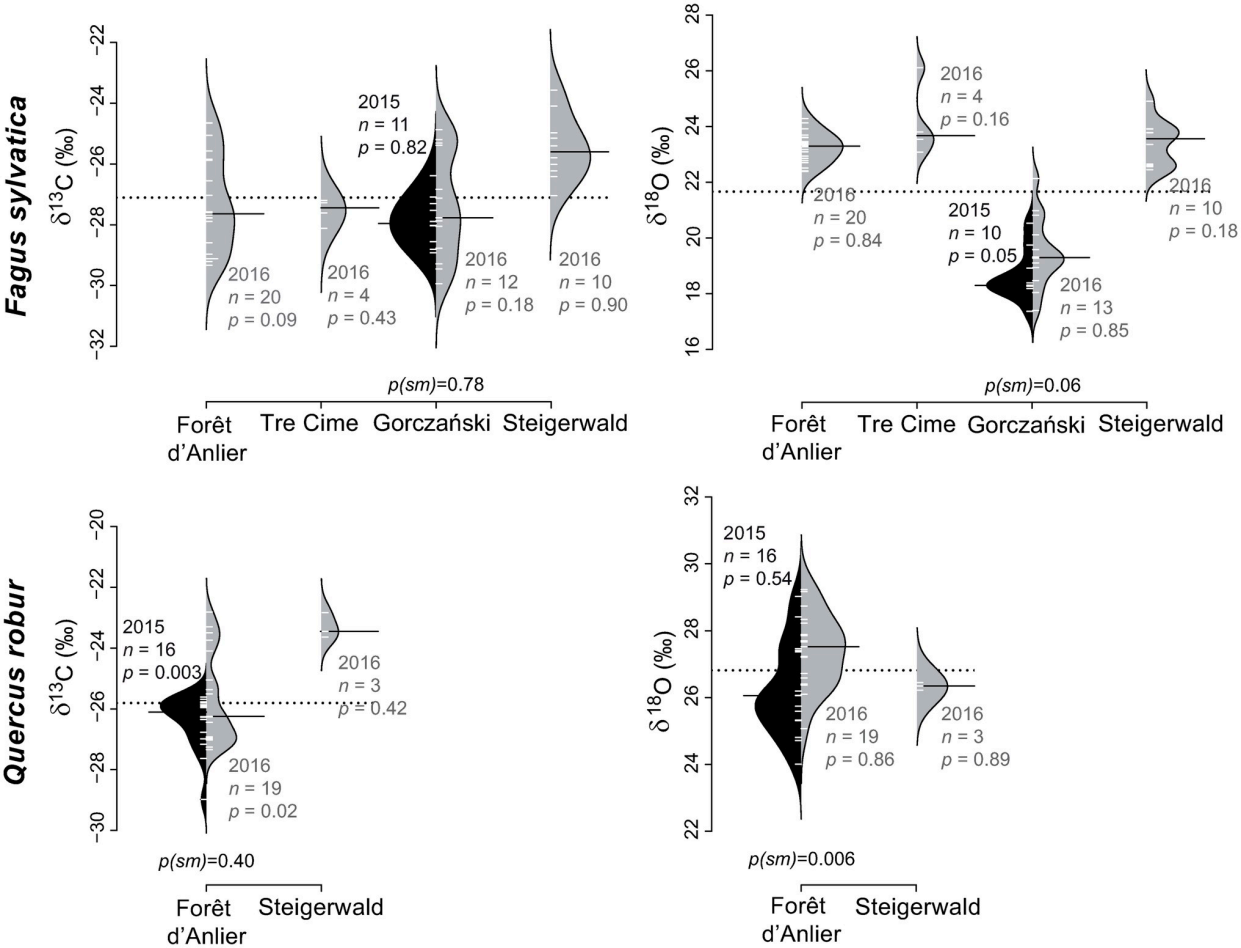
Pollen-isotopes of broad-leaved species flowering April to May: *Fagus sylvatica* and *Quercus robur*. The broad-leaved species *Fagus sylvatica* and *Quercus robur* were sampled at two to four locations. The bean plots show δ^13^C_*pollen*_ values (left) and δ^18^O_*pollen*_ values (right) of 2015 (black) and 2016 (grey). *n* indicates the number of individuals. *p-*values indicate whether the pollen-isotope values of a single year are normally distributed (sign. level = 0.05), whereas *p(sm)* represents the probability for equal medians in samples of two consecutive years. The dotted line represents the mean over all localities and both years. The means of each sampling are indicated by a black bar.

The range of *F*. *sylvatica* mean δ^13^C_*pollen*_ values is -27.9‰ to -25.5‰ (2.4‰) and the values are normally distributed with similar medians for both years at the site Gorczański (Table 3, Fig 6). Mean δ^13^C_*pollen*_ values of *Q*. *robur* range from -26.4‰ to -23.3‰ (3.1‰). The δ^13^C_*pollen*_ values are not normally distributed at Forêt d’Anlier (2015: *p* = 0.003; 2016: *p* = 0.02), but the carbon isotope values of both years are similar at this site (*p(sm)* = 0.40).

#### Flowering period May to June: *Pinus sylvestris* and *Picea abies*

**Fig 7:**
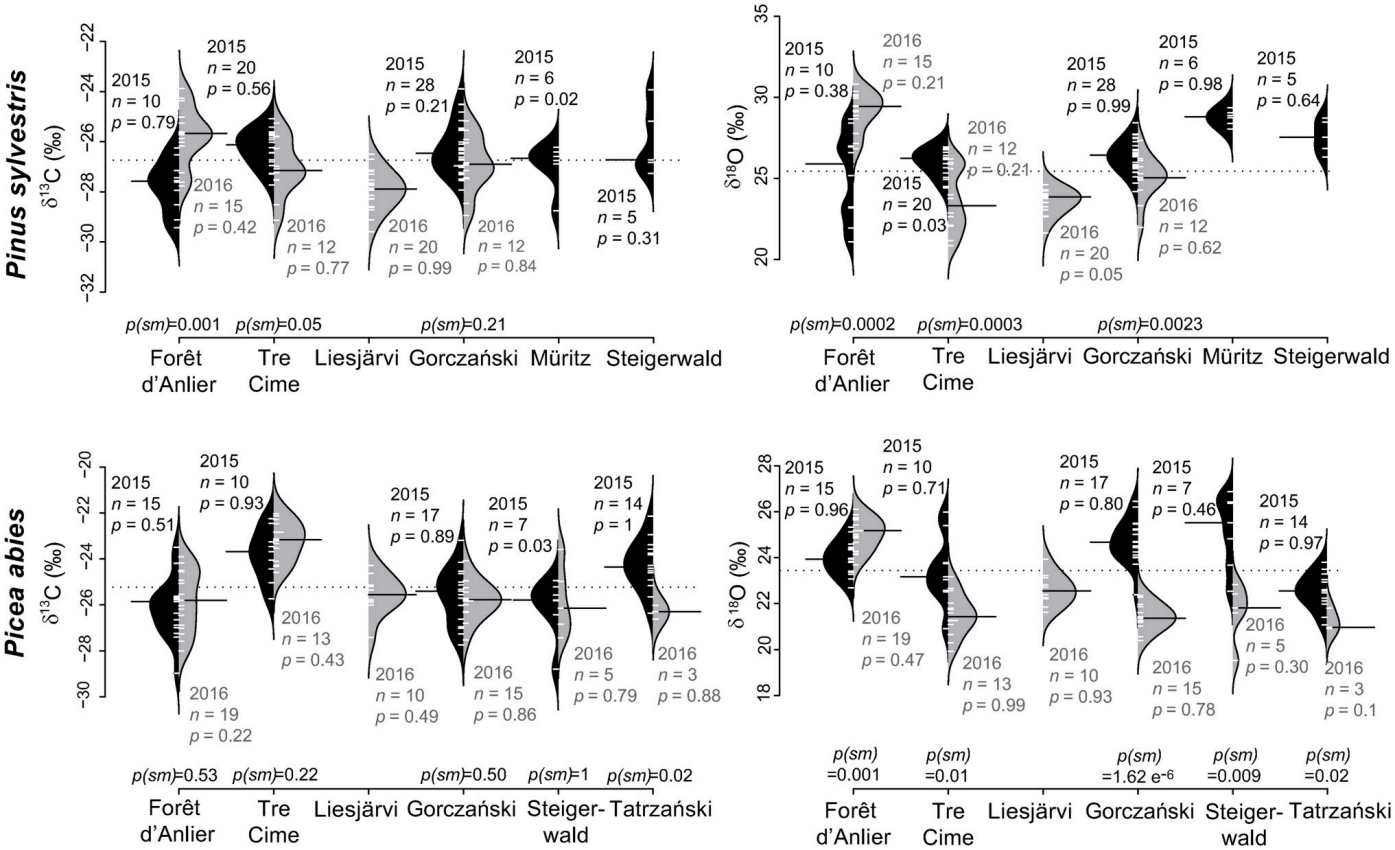
Pollen-isotopes of coniferous species flowering May to June: *Pinus sylvestris* and *Picea abies*. *Pinus sylvestris* and *Picea abies* were sampled at six locations. The bean plots show δ^13^C_*pollen*_ values (left) and δ^18^O_*pollen*_ values (right) of 2015 (black) and 2016 (grey). *n* indicates the number of individuals, *p* represents the probability of normally distributed pollen-isotopes within one year and *p(sm)* indicates the probability for similar medians in samples of two consecutive years. The dotted line represents the mean over all localities and both years. The means of each sampling are indicated by a black bar.

*Pinus sylvestris* mean δ^13^C_*pollen*_ values range between -27.9‰ and -26.0‰ (1.9‰; Table 3, Fig 7) and the values are normally distributed with one exception at Müritz (2015; *p* = 0.02). Medians of both years are mostly comparable, only Forêt d’Anlier yields statistically distinct medians at *p(sm)* = 0.001. The mean δ^13^C_*pollen*_ values of *P*. *abies* range from -26.3‰ to -23.2‰ (3.1‰; Table 3) and only the samples from Steigerwald (2015; *p* = 0.03) are not normally distributed. Between-year equal medians are present for all locations except Tatrzański (*p(sm)* = 0.02).

### Intra-tree variability, intra-annual variability and variability with elevation of δ^13^C_*pollen*_

#### Intra-tree variability of δ^13^C_*pollen*_

64% of δ^13^C_*pollen*_ values of samples taken at lower branches of the broad-leaved species *A*. *pseudoplatanus A*. *glutinosa* and *C*. *avellana* are more negative in comparison to the branches higher up in the same individuals (Fig 3, Table 4). The isotopic values of the samples taken at different positions within the canopy are mostly ranging within one or two standard deviations from the mean isotope value of the tree. δ^13^C_*pollen*_ values from pollen growing at the east side of a tree are likely to be higher, whereas values of the west tend to show lower δ^13^C_*pollen*_ values in comparison to the mean isotope value of the trees. Deviations from the mean at northern and southern positions appear to be species-specific. The δ^13^C_*pollen*_ value of *A*. *pseudoplatanus* is lower in the North (-0.7‰ from the mean value) and *A*. *glutinosa* yields lower values in the North in two out of three samples (Table 4). The carbon isotope depletion in the North averages at -0.4‰ for *A*. *glutinosa*. In contrast, *C*. *avellana* exhibits higher pollen-isotope values in the North (+0.5‰) compared to the intra-tree average of this species. Twenty samples taken at different positions within the canopy of a single *P*. *abies* tree show higher δ^13^C_*pollen*_ values at eastern and western positions and lower values at the southern exposition (Table 5). The analysis of 34 individual inflorescences from low and high positions demonstrates the intra-branch variability in pollen-isotopes of six neighbouring *P*. *sylvestris* trees (Table 6). δ^13^C_*pollen*_ values from the same branch of one individual tree differ in a range of 0‰ to 1.2‰ (Table 6). The average δ^13^C_*pollen*_ difference between inflorescences from the same branch is 0.3‰.

**Table 4:** Isotopic deviation of δ^13^C_*pollen*_ values and δ^18^O_*pollen*_ values between different sampling heights and cardinal directions.

ID	direct.	pos.	δ^13^C	δ^18^O	var. δ^13^C	var. δ^18^O	av. δ^13^C	av. δ^18^O		de. δ^13^C			de. δ^18^O	
			(‰)	(‰)	(L-H)	(L-H)	(‰)	(‰)		(‰)			(‰)	
*Acer*_1 (Steigerwald)	North	L	-25.7	25.8	-	-	-25.7	25.8						
		H	-	-										
	South	L	-24.8	25.1	-0.4	0.2	-24.6	25.0		N- 0.7			N +0.4	
		H	-24.3	24.8					W +0.0	**-24.8** (av._*tree*_)	E +0.3	W -0.1	**25.4** (av._*tree*_)	E +0.3
	East	L	-25.2	25.3	-1.1	-1.0	-24.6	25.7						
		H	-24	26.2						S +0.4			S -0.5	
	West	L	-25.4	25.0	-1	-0.8	-24.9	25.3						
		H	-24.4	25.7										
*Alnus*_1 (F. d’Anlier)	North	L	-	-	-	-	-26.4	21.4						
		H	-26.4	21.4										
	South	L	-	-	-	-	-25.8	21.9		N +0.3			N -0.2	
		H	-25.8	21.9					W -1.4	**-26.7** (av._*tree*_)	E +0.2	W +0.1	**21.6** (av._*tree*_)	E -0.2
	East	L	-	-	-	-	-26.5	21.4						
		H	-26.5	21.4						S +1.1			S +0.3	
	West	L	-28.1	21.6	-	-	-28.1	21.6						
		H	-	-										
*Alnus*_9 (F. d’Anlier)	North	L	-29.9	21.6	-2.3	-1.3	-28.8	22.3						
		H	-27.6	22.9										
	South	L	-27.6	21.7	-	-	-27.6	21.7		N -0.5			N +0.4	
		H	-	-					W +0.4	**-28.3** (av._*tree*_)	E +0.6	W -0.7	**21.8** (av._*tree*_)	E +0.4
	East	L	-	-	-	-	-29.0	22.2						
		H	-29.0	22.2						S +0.7			S -0.2	
	West	L	-27.5	21.1	0.9	-0.1	-27.9	21.1						
		H	-28.4	21.2										
*Alnus*_11 (Gorczański)	North	L	-28.7	19.0	-0.9	-0.7	-28.3	19.4						
		H	-27.8	19.8										
	South	L	-26.5	18.5	-0.6	-0.2	-26.2	18.6		N -0.9			N +0.3	
		H	-25.9	18.7					W -0.1	**-27.3** (av._*tree*_)	E -0.2	W +0.3	**19.1** (av._*tree*_)	E -0.1
	East	L	-27.6	19.1	-	-	-27.6	19.1						
		H	-	-						S +1.2			S -0.5	
	West	L	-27.6	19.5	-0.3	0.2	-27.5	19.4						
		H	-27.3	19.3										
*Corylus*_11 (Gorczański)	North	L	-24.6	19.1	0.8	-0.3	-25.0	19.3						
		H	-25.3	19.4										
	South	L	-25.4	18.9	0.9	-0.3	-27.8	19		N +0.7			N -0.0	
		H	-26.2	19.1					W +0.1	**-25.1** (av._*tree*_)	E +1.4	W +0.1	**19.3** (av._*tree*_)	E +0.3
	East	L	-24.2	20.1	0.1	1.0	-24.3	19.6						
		H	-24.3	19.1						S -2.2			S -0.3	
	West	L	-25.1	19.3	0.8	-0.1	-25.5	19.4						
		H	-25.9	19.4										
*Corylus*_19 (Gorczański)	North	L	-27.0	20.3	-2.5	-0.5	-25.7	20.5						
		H	-24.5	20.8										
	South	L	-27.5	18.9	0.3	-0.1	-27.6	18.9		N +1.4			N +1.1	
		H	-27.8	19.0					W -1.0	**-27.2** (av._*tree*_)	E -0.0	W -0.4	**19.4** (av._*tree*_)	E -0.2
	East	L	-27.7	19.6	-1.1	0.7	-27.2	19.2						
		H	-26.6	18.9						S -0.5			S -0.5	
	West	L	-28.6	18.4	-0.9	-1.2	-28.1	19.0						
		H	-27.7	19.6										
*Corylus*_7 (F. d’Anlier)	North	L	-29.6	23.4	-0.9	-0.1	-29.2	23.5						
		H	-28.7	23.5										
	South	L	-30.4	22.2	-1.1	-0.1	-29.8	22.3		N +0.2			N +1.0	
		H	-29.3	22.3					W -0.5	**-29.2** (av._*tree*_)	E +0.9	W -1.2	**22.7** (av._*tree*_)	E +0.4
	East	L	-29.9	23.0	-2.9	0.0	-28.4	23.0						
		H	-27.0	23.0						S -0.5			S -0.3	
	West	L	-29.8	21.4	-	-	-29.8	21.4						
		H	-	-										
*Corylus*_21 (F. d’Anlier)	North	L	-29.0	23.1	-0.7	0.1	-28.6	23.1						
		H	-28.3	23.0										
	South	L	-27.0	22.3	1.5	-0.5	-27.7	22.5		N -0.4			N +0.5	
		H	-28.5	22.7					W -1.3	**-28.4** (av._*tree*_)	E +1.3	W -0.3	**22.6** (av._*tree*_)	E -0.2
	East	L	-	-	-	-	-26.9	22.4						
		H	-26.9	22.4						S +0.4			S -0.1	
	West	L	-29.2	22.5	0.5	0.4	-29.5	22.3						
		H	-29.7	22.1										

Analysis of samples from eight individual trees of three species (*Acer pseudoplatanus*, *Alnus glutinosa* and *Corylus avellana*) taken at each cardinal direction and from two different positions on each tree. ID = individual identification (including species, number and site); direct. = cardinal direction; pos. = position on the tree (L = low; H = high); var. = variance; av. = average; de. = deviation from the mean value. The mean isotope values of the tree are noted in the black box (av._*tree*_).

**Table 5:** Intra-tree analysis of a single *Pices abies* tree from Tre Cime, Italy.

ID	direct.	pos.	inf._ID	δ^13^C	δ^18^O	av. δ^13^C	av. δ^18^O	var. δ^13^C	var. δ^18^O			
				(‰)	(‰)	(‰)	(‰)	(L-H)	(L-H)			
*Picea*_12 (Tre Cime)	South	L	-	-22.4	23.4	-22.5	23.1	-0.1	1.9			
			S_low_1	-22.5	23.4						**de. δ**^**13**^**C**	
			S_low_2	-22.8	21.6						**(‰)**	
			S_low_3	-22.2	23.9						N	
		H	-	-22.5	21.1	-22.3	21.3			W +0.1	**-22.2** (av._*tree*_)	E +0.2
			S_high_1	-22.3	20.6							
			S_high_2	-22.8	21.9						S -0.4	
			S_high_3	-21.7	21.5							
	East	L	-	-22.6	22.9	-22.5	23.2	-1.0	-0.4			
			E_low_1	-21.3	22.6							
			E_low_2	-23.6	24.2							
		H	-	-21.2	23.5	-21.5	23.7				**de. δ**^**18**^**O**	
			E_high_1	-21.8	23.7						**(‰)**	
			E_high_2	-21.4	23.8						N	
	West	L	-	-22.3	23	-21.7	23.1	0.6	-0.5	W +0.3	**22.9** (av._*tree*_)	E +0.5
			W_low_1	-21.8	22.6							
			W_low_2	-21.1	23.6						S -0.8	
		H	-	-21.8	23.2	-22.3	23.5					
			W_high_1	-22.4	24.3							
			W_high_2	-22.8	23							

Analysis of samples from three cardinal directions (south, east and west; the northern side was not flowering), different sampling heights (low/high) and the comparison of individual inflorescences from each sampling position. ID = individual identification (including species, number and site); direct. = cardinal direction; pos. = position on the tree (L = low; H = high); inf._ID = inflorescence identification per branch; var. = variance; av. = average; de. = deviation from the mean value. The mean isotope values of the tree are noted in the black box (av._*tree*_).

**Table 6:** Intra-tree variability of δ^13^C_*pollen*_ and δ^18^O_*pollen*_ values from single inflorescences of six *Pinus sylvestris* trees.

Tree ID	Position	Branch ID	Inf. ID	δ^13^C	δ^18^O	de. δ^13^C	de. δ^18^O
				(‰)	(‰)	(‰)	(‰)
*Pinus* 1	L	low_1	1	-25.3	27.0	0.7	0.8
	L		2	-26.0	27.8		
	L	low_2	1	-24.8	28.0	0.4	0.4
	L		2	-25.2	28.4		
	H	high_1	1	-25.7	27.2	0.1	1.4
	H		2	-25.6	28.6		
	H	high_2	1	-25.4	27.3	0.2	0.1
	H		2	-25.2	27.2		
*Pinus* 2	L	low_1	1	-23.7	26.4	0.0	0.2
	L		2	-23.7	26.6		
	L	low_2	1	-24.0	26.5	0.3	0.7
	L		2	-23.7	27.2		
	H	high_1	1	-24.5	26.1	0.1	0.2
	H		2	-24.4	26.3		
	H	high_2	1	-23.8	26.4	0.0	0.7
	H		2	-23.8	27.1		
*Pinus* 3	L	low_1	1	-23.3	27.4	1.2	0.1
	L		2	-24.5	27.5		
	L	low_2	1	-24.7	26.7	0.2	0.2
	L		2	-24.9	26.9		
	H	high_1	1	-24.8	26.9	0.0	0.8
	H		2	-24.8	27.7		
	H	high_2	1	-25.0	26.6	0.2	0.4
	H		2	-25.2	26.2		
*Pinus* 4	L	low_1	1	-25.4	27.6	0.8	0.5
	L		2	-24.6	27.1		
	H	high_1	1	-24.6	27.5	0.0	0.3
	H		2	-24.6	27.8		
*Pinus* 5	L	low_1	1	-26.4	26.3	0.2	0.7
	L		2	-26.6	27.0		
*Pinus* 6	L	low_1	1	-27.8	27.2	0.6	0.3
	L		2	-27.2	26.9		
	H	high_1	1	-27.0	27.4	0.9	0.1
	H		2	-27.9	27.3		
**Average:**						**0.3**	**0.5**

The samples of six individual *Pinus sylvestris* trees from the same location represent single inflorescences from different positions (low/high) on the tree. The isotopic difference between the inflorescences shows the variability of δ^13^C_*pollen*_ and δ^18^O_*pollen*_ values on branches and within trees at a high resolution. Tree ID = individual identification, (including species, number and site); Position = position on the tree (L = low; H = high); Branch ID = branch identification at each tree; Inf. ID = inflorescence identification of each branch; de. = deviation from the mean value.

#### δ^13^C_*pollen*_ values at different stages of pollen maturation

**Fig 8:**
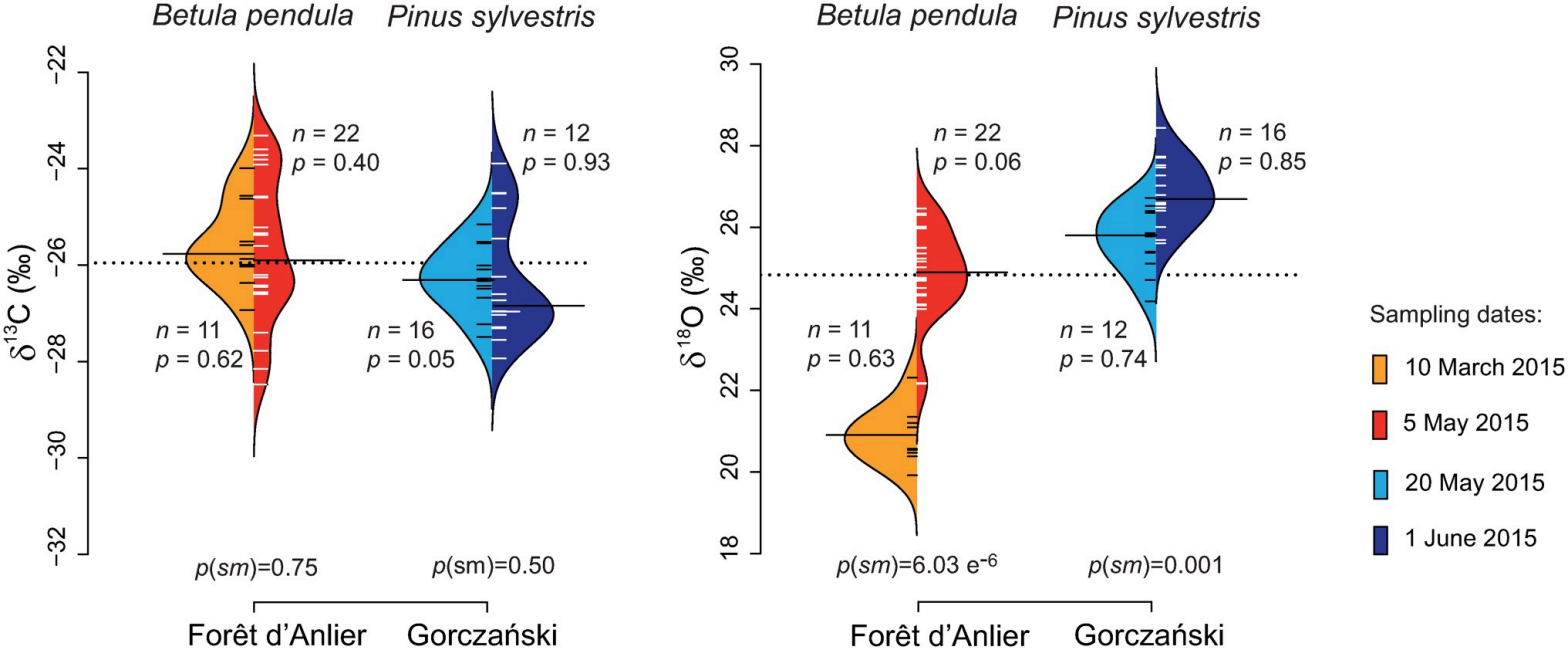
Intra-annual comparison of δ^13^C_*pollen*_ and δ^18^O_*pollen*_ values of *Betula pendula* (Forêt d’Anlier) and *Pinus sylvestris* (Gorczański). Both species were sampled twice in 2015. The bean plots show δ^13^C_*pollen*_ values (left) and δ^18^O_*pollen*_ values (right). Colours indicate the sampling date (orange = 10 March 2015; red = 5 May 2015; light blue = 20 May 2015; dark blue = 1 June 2015). *n* is the number of individuals sampled. The *p-*values indicate whether the pollen-isotope values of one year are normally distributed (sign. level = 0.05), whereas *p(sm)* represents the probability for equal medians in samples of the same year. The dotted line represents the mean over all localities and both years. The means of each sampling are indicated by a black bar.

The δ^13^C_*pollen*_ values of *B*. *pendula* collected in March (-25.6‰) and May (-25.7‰) 2015 and of *P*. *sylvestris* collected in May (-26.3‰) and June (-26.3‰) 2015 are normally distributed and the statistical test reveals similar medians of the isotope values for both samplings of *B*. *pendula* and *P*. *sylvestris* (Fig 8).

#### Isotope variability of δ^13^C_*pollen*_ with elevation

**Fig 9:**
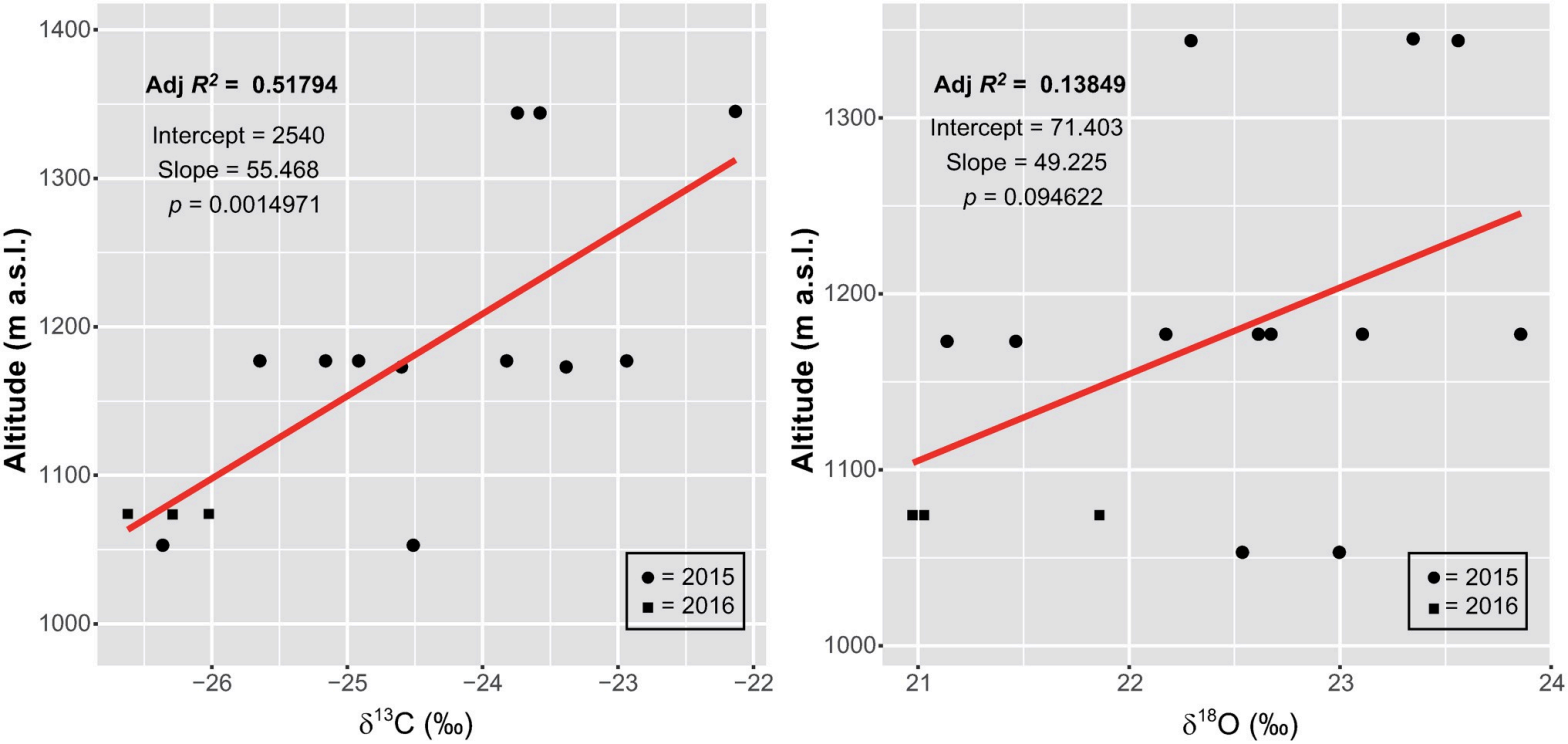
300 m altitudinal transect of *Picea abies* at Tatrzański Park Narodowy. The plots show the linear regression analysis of δ^13^C_*pollen*_ values and altitude (left) and δ^18^O_*pollen*_ values and altitude (right). Samples were taken in 2015 (black dots) and 2016 (black squares). The red line indicates the slope of the linear regression model. Adjusted *R*^*2*^ indicates the proportion of variance explained by the linear association of isotopes and elevation.

The δ^13^C_*pollen*_ values of *P*. *abies* at Tatrzański Park Narodowy increase with altitude from -26.6‰ (1053 m a.s.l) up to -22.1‰ (1344 m a.s.l.) (Fig 9). The coefficient *R*^*2*^ of 0.52 reveals a weak linear correlation with elevation.

### Stepwise regression analysis for δ^13^C_*pollen*_

#### By species

The most relevant factor influencing δ^13^C_*pollen*_ values for several species is *site* with very high probability values of Prob > F = < 0001* (Table 7; S1 Dataset). The *year* of sampling influenced δ^13^C_*pollen*_ of *B*. *pendula*, *C*. *avellana* and *Q*. *robur*. The factor *soil* and the *maturity* of the pollen are important for *A*. *glutinosa* and *P*. *sylvestris*. The factors *proximity to water* and *water classification* occur only once in the analytical outcome.

**Table 7:** Environmental factors affecting stable pollen-isotope composition for each species.

Plant species	Influencing factor on δ^13^C_*pollen*_	Prob > F	Influencing factor on δ^18^O_*pollen*_	Prob > F
*A*. *pseudoplatanus*	proximity to water	0.0266*	site {GOR-FAN&DOL&STE}	<.0001*
			site {DOL&STE-FAN}	0.0043*
*A*. *glutinosa*	soil	0,0097	year	0.0034*
	maturity	0.0012*	site {GOR-FAN&MUR&STE}	<.0001*
			site {FAN-MUR&STE}	0.0162*
			site {MUR-STE}	0.0009*
			maturity	<.0001*
*B*. *pendula*	year	<.0001*	year	0.0165*
			month {mar-apr&may}	<.0001*
*C*. *betulus*	water classification	0.0186*	proximity to water	0.0048*
*C*. *avellana*	year	0.0047*	site {GOR-FAN&STE&MUR}	<.0001*
	site {FAN—GOR&STE&MUR}	<.0001*	site {FAN-STE&MUR}	0.0003*
*F*. *sylvatica*	site {GOR&TRE&FAN—STE}	0.0001*	site {GOR-FAN&STE&TRE}	<.0001*
			maturity	<.0001*
*P*. *abies*	site {FAN&STE&GOR&LIE -TAT&TRE}	<.0001*	year	<.0001*
	site {TAT-TRE}	0.0022*	site {TRE&TAT&LIE&GOR-STE&FAN}	0.0055*
			site {TRE&TAT-LIE}	0.0068*
			water_classification	<.0001*
*P*. *sylvestris*	maturity	0.0009*	site {LIE&TRE&GOR—STE&FAN&MUR}	<.0001*
	soil	0.0018*	month	0.0124*
			maturity	<.0001*
*Q*. *robur*	year	0.0240*	year	0.0146*

Relevant environmental impact factors on δ^13^C_*pollen*_ and δ^18^O_*pollen*_ values for each species. Prob > F gives the probability value after Levene´s test [46]. The factor *site* groups locations by similarity. Abbreviations for locations: FAN (Forêt d’Anlier); GOR (Gorczański); MUR (Müritz); STE (Steigerwald); TAT (Tatrzański); TRE (Tre Cime); LIE (Liesjärvi).

#### By site

The site-specific statistical evaluation of the deviation of δ^13^C_*pollen*_ values from the means reveals the factor *species* as the most important one (Table 8; S2 Dataset). Species are often hierarchically grouped by statistical similarity. The *year* of sampling influences the pollen variability at two sites (Forêt d’Anlier and Gorczański) and also *maturity* occurs twice (Tre Cime and Tatrański). *Soil* (Gorczański) and *altitude* (Tatrzański) are once listed as influencing factors.

**Table 8:** Environmental factors affecting stable pollen-isotope composition at each site.

Site	Influencing factor on δ^13^C_*pollen*_	Prob > F	Influencing factor on δ^18^O_*pollen*_	Prob > F
Parc Naturel Forêt d’Anlier	species {*A*.*glutinosa* & *C*.*avellana*–*F*.*sylvatica* & *P*.*sylvestris* & *Q*. *robur* & *P*.*abies* & *C*.*betulus* & *A*.*pseudoplatanus* & *B*. *pendula*}	<.0001*	species {*A*.*glutinosa* & *C*.*avellana* & *F*.*sylvatica* & *B*.*pendula* & *P*.*abies*–*A*.*pseudoplatanus* & *C*.*betulus* & *Q*.*robur* & *P*.*sylvestris* }	<.0001*
	species {*F*.*sylvatica* & *P*.*sylvestris*—*Q*.*robur* & *P*.*abies* & *C*.*betulus* & *A*.*pseudoplatanus* & *B*.*pendula*}	<.0001*	species {*A*.*glutinosa* & *C*.*avellana*—*F*.*sylvatica* & *B*.*pendula* & *P*.*abies* }	0.0189*
	species {*Q*.*robur* & *P*.*abies* & *C*.*betulus*—*A*.*pseudoplatanus* & *B*.*pendula*}	0.0004*	species {*F*.*sylvatica* & *B*.*pendula–P*.*abies*}	0.0002*
	year	0.0060*	species {*F*.*sylvatica*—*B*.*pendula*}	<.0001*
			species {*A*.*pseudoplatanus* & *C*.*betulus* & *Q*.*robur*—*P*.*sylvestris*}	<.0001*
			species {*A*.*pseudoplatanus*–*C*.*betulus* & *Q*.*robur*}	0.0042*
			year	0.0004*
			month	<.0001*
			maturity	0.0017*
			water classification	0.0013*
PN Tre Cime	species {*F*.*sylvatica* & *P*.*sylvestris* & *A*.*pseudoplatanus—B*.*pendula* & *P*.*abies*}	<.0001*	species {*P*.*abies*—*A*.*pseudoplatanus* & *B*.*pendula* & *F*.*sylvatica* & *P*.*sylvestris*}	<.0001*
	species {*B*.*pendula*- *P*.*abies*}	0.0010*	year	<.0001*
	maturity	0.0085*		
Liesjärvi k.	species	<.0001*	species	0.0028*
Gorczański Park Narodowy	species {F.sylvatica & *C*.*avellana* & *A*.*glutinosa* & *P*.*sylvestris*—*P*.*abies* & *A*.*pseudoplatanus*}	<.0001*	species {*A*.*pseudoplatanus* & *A*.*glutinosa* & *C*.*avellana* & *F*.*Sylvatica*—*P*.*abies* & *P*.*sylvestris*}	<.0001*
	year	0.0096*	species {*P*.*abies*—*P*.*sylvestris*}	<.0001*
	soil	<0.0006*	altitude	0.0204*
			month	0.0021*
Müritz NP	species {*A*.*glutinosa*—*P*.*sylvestris* & *C*.*avellana* & *B*.*pendula*}	<.0001*	species {*A*.*glutinosa* & *C*.*avellana* & *B*.*pendula*—*P*.*sylvestris*}	<.0001*
			species {*A*.*glutinosa*—*C*.*avellana* & *B*.*pendula*}	<.0001*
Steigerwald National Park	species {*C*.*avellana* & *A*.*glutinosa* & *P*.*sylvestris* & *P*.*abies* & *C*.*betulus* & *F*.*sylvatica*–*A*.*pseudoplatanus* & *Q*.*robur* & *B*.*pendula*}	<.0001*	species {*A*.*glutinosa* & *F*.*sylvatica* & *C*.*avellana* & *P*.*abies* & *A*.*pseudoplatanus* & *B*.*pendula*—*C*.*betulus* & *Q*.*robur* & *P*.*sylvestris*}	0.0017*
			species {*A*.*glutinosa* & *F*.*sylvatica* & *C*.*avellana* & *P*.*abies*–*A*.*pseudoplatanus* & *B*.*pendula*}	<.0001*
			year	<.0001*
			altitude	0.0028*
Tatrzański PN	altitude	0.0026*	year	0.0127*
	maturity	0.0196*		

Relevant environmental factors on δ^13^C_*pollen*_ values and δ^18^O_*pollen*_ for each site. Prob > F gives the probability value after Levene´s test [46]. Hierarchical clusters follow statistical similarity of the δ^13^C_*pollen*_ and δ^18^O_*pollen*_ values within the factor *species*. The significance level lies at 0.05.

### δ^18^O_*pollen*_ values of broad-leaved and coniferous tree species

#### Flowering period January to March: *Alnus glutinosa* and *Corylus avellana*

Mean δ^18^O_*pollen*_ values of *A*. *glutinosa* range from 18.1‰ to 22.9‰ (4.8‰; Table 3). Values of each year are normally distributed (Fig 4). However, their medians are statistically distinct for site Gorczański at *p(sm)* = 0.0002. Mean *C*. *avellana* δ^18^O_*pollen*_ values range from 18.5‰ to 23.9‰ (5.4‰, Table 3). The pollen-isotope values are normally distributed but years 2015 and 2016 yield statistically distinct medians for Forêt d’Anlier.

#### Flowering period April to May: *Acer pseudoplatanus*, *Betula pendula*, *Carpinus betulus*, *Fagus sylvatica* and *Quercus robur*

Mean δ^18^O_*pollen*_ values of *A*. *pseudoplatanus* range between 18.0‰ and 25.7‰ (7.7‰; Table 3, Fig 5). Values of each year are normally distributed except for site Tre Cime (2015, at *p* = 0.04). The statistical test reveals similar medians between years. Mean *B*. *pendula* δ^18^O_*pollen*_ values range from 23.5‰ to 24.8‰ (1.3‰) and are normally distributed with one exception (Forêt d’Anlier 2015, *p* = 0.004; Fig 5). The δ^18^O_*pollen*_ values of 2015 and 2016 yield similar medians (*p(sm)* = 0.75). Mean δ^18^O_*pollen*_ values of *C*. *betulus* range from 26.1‰ to 26.3‰ (0.2‰; Table 3) and are also normally distributed (Fig 5). The comparatively low mean δ^18^O_*pollen*_ values of *F*. *sylvatica* range from 18.6‰ to 24.1‰ (5.5‰; Table 3) and the values are normally distributed with similar medians for both years from Gorczański (Fig 6). Mean *Q*. *robur* δ^18^O_*pollen*_ values range between 26.2‰ and 27.4‰ (1.2‰; Table 3) and are normally distributed but statistically distinct between both years at *p(sm)* = 0.006 (Fig 6).

#### Flowering period May to June: *Pinus sylvestris* and *Picea abies*

Mean δ^18^O_*pollen*_ values of *P*. *sylvestris* range from 23.6‰ to 29.2‰ (5.6‰; Table 3) and the values are normally distributed except for the samples at Tre Cime collected in 2015 (*p* = 0.03). All comparisons between the two vegetation periods are statistically distinct. Mean *P*. *abies* δ^18^O_*pollen*_ values range between 21.3‰ and 25.2‰ (3.9‰; Table 3). The values are normally distributed within each sampling (Fig 7) but comparisons between 2015 and 2016 are statistically distinct for all sites.

### Intra-tree variability, intra-annual variability and variability with elevation of δ^18^O_*pollen*_

#### Intra-tree variability of δ^18^O_*pollen*_

68% of the δ^18^O_*pollen*_ values of samples from *A*. *pseudoplatanus*, *A*. *glutinosa* and *C*. *avellana* taken at the lower branches are more negative in comparison to the values of the higher branches from the same individual (Fig 3, Table 4). The δ^18^O_*pollen*_ values were often lower at southern and western positions, whereas values from the northern position seem to be generally higher for all species (Table 4). Twenty samples taken at different positions within the canopy of a *P*. *abies* tree show higher δ^18^O_*pollen*_ at eastern and western positions, and lower values at the southern exposition (Table 5). Similar to the broad-leaved species, δ^18^O_*pollen*_ values from lower branches of *P*. *abies* tend to be lower than those from higher branches (Table 5). The analysis of individual inflorescences demonstrates an intra-branch variability ranging between 0‰ and 1.4‰ (Table 6). The average deviation for inflorescences from the same branch is 0.5‰ for δ^18^O_*pollen*_.

#### δ^18^O_*pollen*_ values at different stages of pollen maturation

The δ^18^O_*pollen*_ values of *B*. *pendula* are normally distributed (Fig 8), but the medians are statistically distinct. The mean δ^18^O_*pollen*_ values increase by 4‰ within 11 weeks (from 20.9‰ on 10 March to 24.9‰ on 5 May). δ^18^O_*pollen*_ values of *P*. *sylvestris* are also normally distributed for each sampling (Fig 8). However, they increase by 1.2‰ within 12 days: δ^18^O_*pollen*_ were at 25.7‰ on 20 May and at 26.9‰ on 1 June and the medians are statistically distinct.

#### Isotope variability of δ^18^O_*pollen*_ with elevation

δ^18^O_*pollen*_ values of *P*. *abies* at Tatrzański Park Narodowy (Fig 9) are scattered throughout the elevation transect and do not correlate with elevation (*R*^*2*^ = 0.14). The values range from 21.0‰ (1074 m a.s.l.) to 23.9‰ (1177 m a.s.l.).

### Stepwise regression analysis of δ^18^O_*pollen*_

#### By species

δ^18^O_*pollen*_ values were found to be affected by variable factors. The most important factor was *site*, which impacts the δ^18^O_*pollen*_ variability in six species (Table 7). The *year* of sampling influences the δ^18^O_*pollen*_ variability in four species, whereas the *maturity* of the pollen determines the δ^18^O_*polle*_ variability in three species and the factor *month* of sampling occurs twice. The factors *proximity to water* and *water classification* are rarely influential and, if significant, they were species-specific.

#### By site

δ^18^O_*pollen*_ value variability within sites is mostly determined by the factor *species* (Table 8). The *year* of sampling is important in four cases and the *month* of sampling and *altitude* occur twice in the analytical outcome. The factors *maturity* of the pollen and *water classification* are each listed once.

## Discussion

### δ^13^C_*pollen*_ values of broad-leaved species flowering January to March

#### *Alnus glutinosa* and *Corylus avellana*

Like other winter-deciduous tree species, *A*. *glutinosa* and *C*. *avellana* (both family Betulaceae) have a similar timing of leaf unfolding and senescence, i.e. a similar duration of the photosynthetically active period in one year [47–52]. Nonetheless, their δ^13^C_*pollen*_ values are generally low compared to tree species flowering April to June (*A*. *glutinosa*: -28.2‰, *C*. *avellana*: -27.4‰; Table 3). Catkins of both species occur before the leaf buds emerge (Fig 2), thus the pollen can only be polymerized from stored fatty and amino acids, phenols and other precursors of sporopollenin predominately accumulated during previous vegetation periods. *Alnus glutinosa* and *C*. *avellana* pollen are present and fully developed in shape before winter dormancy, but the size of maturing pollen grains increases in early spring until a few days before pollen shedding [53]. This indicates that some biopolymers are still added to the pollen grains and possibly exchanged right before pollination.

Ambient air temperatures shortly before the onset of flowering affect the catkin development of both species and the onset date for trees flowering in early spring is highly variable, even between consecutive years [54]. However, the responsiveness of the plant to favorable weather conditions does not seem to have any marked effect on its δ^13^C_*pollen*_ values. The measurements indicate similar medians for consecutive years for *A*. *glutinosa* (*p(sm)* = 0.26 and 0.17; Fig 4), although flowering started roughly two weeks earlier at all sites sampled in 2016 (personal observation). *Corylus avellana* δ^13^C_*pollen*_ values deviate more strongly between the two years (*p(sm)* = 0.07 and 0.004; Fig 4). But even though both species react differently over two vegetation periods, they show similar patterns between particular sites (Fig 4): The signals of the western sampling location of Forêt d’Anlier are similar to the signals of the eastern sampling location of Gorczański (Fig 1).

Especially noticeable are the low values of δ^13^C_*pollen*_ for *A*. *glutinosa* from Müritz (2016) compared to those of other sites. The mean δ^13^C_*pollen*_ value is -31.0‰ and thus 2.8‰ lower than the average *A*. *glutinosa* pollen-isotope value of -28.2‰ from all sampling sites. However, all values lie within the range of a normal distribution (*p* = 0.81; Fig 4), so we can exclude measurement errors or tree-individual outliers to have caused the observed low mean δ^13^C_*pollen*_ value. Additionally, *C*. *avellana* from the same site and year (Müritz 2016) does not show exceptionally low values in comparison to data of that species measured at other sites. At the moment, we do not have a valid explanation for the unusual deviation of δ^13^C_*pollen*_ values of *A*. *glutinosa* sampled at Müritz (2016).

### δ^13^C_*pollen*_ values of broad-leaved species flowering April to May

#### Acer pseudoplatanus

Mean δ^13^C_*pollen*_ values of *A*. *pseudoplatanus* (Sapindaceae; -25.1‰) are intermediate compared to other broad-leaved species in this study. Depending on location and year of sampling, the δ^13^C_*pollen*_ values of *A*. *pseudoplatanus* are closest to *Q*. *robur* (-25.7‰, Forêt d’Anlier 2016) and *B*. *pendula* (-24.8‰, Tre Cime 2016), but they show no consistent offset or similarity to any other species examined. In general, *Acer* ssp. are not dependent on prevailing spring temperatures to start seasonal development but instead require a specific amount of daylight [55]. Their leaves emerge early in the season and immediately exhibit a high rate of photosynthesis [56]. However, *Acer* ssp. are easily affected by short-term weather events and under unfavourable conditions, their carbon fixation during photosynthesis is rather ineffective, up to 50% less compared to the genus *Quercus* under the same conditions [57]. Additionally, *A*. *pseudoplatanus* is very sensitive to cold air inversions in spring and autumn [58]. Inter-annual variations in leaf senescence are highly correlated with precipitation: in dry years, high respiration rates cause early senescence and even premature leaf fall [59]. Therefore, photosynthetically active periods of *Acer* ssp. are highly variable even between consecutive years. Hence, their pollen-isotope values may be challenging to interpret.

#### *Fagus sylvatica* and *Quercus robur*

δ^13^C_*pollen*_ values of *Q*. *robur* show a mean of -25.1‰, whereas the mean value of *F*. *sylvatica* is 2‰ lower (-27.1‰). Deviations between the species of the family Fagaceae can be explained by plant physiology and individual phenology. Both were examined at two locations in 2016: Forêt d’Anlier and Steigerwald (Fig 6 and Table 3). Catkins of the genera *Quercus* and *Fagus* emerge during the same period in late April or early May, simultaneously with their first leaves and twigs. This happens roughly two weeks before anthesis is complete and the pollen starts to shed [60]. They both carry winter-dormant leaf and flower buds [61], but the spring ontogeny is genus-specific [50].

*Quercus robur* shows a high sensitivity to favourable spring temperatures regarding bud burst, whereas *F*. *sylvatica* needs twelve hours of daylight to start developing [58]. Trees of the genus *Quercus* are slow in leaf unfolding and it takes about two months until full performance of carbon fixation in the leaves [62]. Thus, the length of the vegetation period in which carbon can be fixated and stored out of a positive net productivity from photosynthesis is shorter for *Quercus* in comparison to *Fagus*. In general, warmer spring temperatures tend to increase carbon uptake, whereas warmer summer and autumn temperatures decrease the uptake due to larger respiration rates [63]. The impact of prevailing temperature on pollen-isotope values during different stages of a vegetation period has yet to be examined in detail.

#### *Betula pendula* and *Carpinus betulus*

Mean δ^13^C_*pollen*_ values of *B*. *pendula* (-24.3‰) are on average 1.3‰ less negative than the mean values of *C*. *betulus* (-25.6‰), even though they belong to the same family (Betulaceae), flower during the same period from mid-April to mid-May and prefer similar habitats (Figs 2 and 5; [60]). The total phase of pollen development spans early September to early April, and the size of *B*. *pendula* pollen grains still increases two to three weeks before pollen release [53], which implies a continuous addition and exchange of biomolecules during pollen maturation. Stach et al. [64] reported a positive correlation of aerial pollen counts of *B*. *pendula* with temperature and rainfall of the year before pollination. Thus, plant physiological reactions to prevailing environmental conditions can be assumed for *B*. *pendula*. *Carpinus betulus* is known to be particularly sensitive to frost damage and tends to prolong catkin proliferation when temperatures in spring are low. That affects the positive net productivity of photosynthesis in early spring [65] and leads to a shift of pollen development and maturation, which may have an effect on the δ^13^C_*pollen*_ values.

#### Differences in pollen-isotopes within families and subfamilies

δ^13^C_*pollen*_ deviate much less within one plant family than between families [14]. However, our findings show that this result does not apply to European taxa of the Betulaceae family. Four tree taxa of this family have been examined in this study (*A*. *glutinosa*, *C*. *avellana*, *B*. *pendula* and *C*. *betulus*). Taxonomically and genetically, the family is divided into two subfamilies: Coryloideae (including *Corylus* and *Carpinus*) and Betuloideae (including *Betula* and *Alnus*). Pollen grains of the members within the subfamilies are almost similar in shape and size [66]. However, due to their different flowering periods from January to March (*A*. *glutinosa* and *C*. *avellana*) and April to May (*B*. *pendula* and *C*. *betulus*), they are isotopically very different. We found that isotopic differences within these subfamilies were higher than between-family differences (Table 9; differences within the Betuloideae δ^13^C_*pollen*_ 3.9‰ and within the Coryloideae δ^13^C_*pollen*_ 1.8‰). Analysing all Betulaceae pollen together would imply a loss of information in the isotope signal.

**Table 9:** Mean isotope values of the examined plant families in Europe.

Plant Family	Subfamily	Species	δ^13^C_*pollen*_	δ^18^O_*pollen*_
			(‰)	(‰)
Betulaceae			-26.4 ± 2.3	22.8 ± 2.7
	Betuloideae	*A*. *glutinosa*	-28.2 ± 1.9	21.1 ± 2.1
		*B*. *pendula*	-24.3 ± 1.6	24.1 ± 1.6
	Coryloideae	*C*. *avellana*	-27.4 ± 1.7	21.7 ± 2.2
		*C*. *betulus*	-25.6 ± 1.7	24.5 ± 1.0
Fagaceae			-26.2 ± 1.6	23.5 ± 3.2
Pinaceae			-26.0 ± 1.5	24.5 ± 2.3
Sapindaceae			-25.1 ± 1.5	22.4 ± 2.5

Mean δ^13^C_*pollen*_ and δ^18^O_*pollen*_ pollen-isotope values and standard deviations of the plant families and of the species within the two subfamilies of the Betulaceae. Mean values for each species include all sites and samplings of 2015 and 2016.

### δ^13^C_*pollen*_ values of coniferous trees flowering May to June

#### *Picea abies* and *Pinus sylvestris*

Even though both species of the family Pinaceae prefer similar habitats [67], the mean carbon isotope offset between the two species is 1.5‰. The mean δ^13^C_*pollen*_ value of *P*. *abies* is -25.3‰ (2015: -25.2‰; 2016: -25.4‰), that of *P*. *sylvestris* lies at -26.8‰ (2015: -26.6‰; 2016: -26.9‰). Thus, analysing bulk coniferous pollen would reduce the environmental information incorporated in the plant material. However, comparison of the mean δ^13^C_*pollen*_ values of *P*. *sylvestris* and *P*. *abies* within the sampling sites reveals almost equal signals for both species between 2015 and 2016. Between-year mean δ^13^C_*pollen*_ values of *P*. *abies* deviate by 0.2‰ and that of *P*. *sylvestris* by 0.3‰. Schwarz [13] reported low δ^13^C_*pollen*_ ranges between four consecutive years for *Pinus retinosa* (0.53‰) and between three years for *Pinus strobus* (1.03‰). Carbon molecules (mostly soluble sugars from the previous vegetation periods) can be allocated in the individuals [68] and are remobilized directly after the resumption of growth in spring [13, 69]. Thus, the usage of stored carbon as basic modules for the pollen might compensate seasonal variations in the δ^13^C_*pollen*_. Hence, between-year median δ^13^C_*pollen*_ values (*p(sm)*; Fig 7) of most sites cannot be statistically distinguished for both species. The stored carbon differs in age between 0.7 years [70] and up to ten years [68].

Tree growth and photosynthetic activity of *P*. *sylvestris* starts approximately 40 days prior to budburst [71]. *Pinus sylvestris* δ^13^C_*pollen*_ values are found to correlate with temperature four to six weeks prior to pollen release [25]. Hence, it can be assumed that the plant uses newly fixated carbon in substantial portions to finish pollen maturation. There is no support for a correlation between North American *Pinus*-species δ^13^C_*pollen*_ and temperature [13]. Thus, this correlation might be species-specific to *P*. *sylvestris* [22].

Intra-site pollen-isotope variability of the δ^13^C_*pollen*_ values of *P*. *abies* range between 0.6‰ and 5.5‰ and that of *P*. *sylvestris* range between 2.5‰ and 4.0‰. Factors determining the intra-site isotope variability of *C*. *atlantica* and herbaceous species are microclimate, physiological differences between the individuals, water and nutrient availability and the number of trees in the direct vicinity [15, 18]. These factors may also account for the isotope variability of *P*. *abies* and *P*. *sylvestris*. Compared to *P*. *abies*, *P*. *sylvestris* has a broader physiological tolerance range to a variety of environmental conditions [67, 72]. Hence, with larger plasticity, physiological reactions turn out smaller and their pollen-isotope values fluctuate less within one location. In general, the pollen-isotope ranges of both species are broader in pollen sampled in 2015 than in samples of 2016.

### Intra-tree variability of δ^13^C_*pollen*_

δ^13^C_*pollen*_ values vary between 1.1‰ and 3.5‰ within an individual tree (Table 4). In most cases the values are higher at the eastern exposed side than at the southern and western sides. With less insolation, open stomata do not discriminate as much against the heavy aerial δ^13^C isotopes [73]. That phenomenon is also expressed by circumferential variations of 1–3‰ in leaf-tissue δ^13^C [73] and Leavitt [74] mentioned an average of 0.5–1.5‰ deviation from the mean within a circumferential tree ring cellulose analysis. In addition, the difference between mean δ^13^C_*pollen*_ values can be up to 3‰ within a height difference of 6 m (Table 4). For 64% of the individuals, δ^13^C_*pollen*_ values of the lower samples of a tree are lower compared to the upper ones (Table 4). These results agree with Schleser [73] who described an enrichment of ^13^C in leaf-tissue of 1–4‰ from bottom to top within one tree. The intra-tree variability between several inflorescences within one branch can be as high as 1.2‰ for δ^13^C_*pollen*_ (Table 6). However, average differences of pollen-isotope values in neighbouring inflorescences of *P*. *sylvestris* are as little as 0.3‰ (δ^13^C_*pollen*_, Table 6).

Due to different carbon and oxygen sources the δ^13^C_*pollen*_ shows a higher intra-tree variability than δ^18^O_*pollen*_ (Table 6). Carbon is fixated in the leaves as the product of photosynthesis. The rate of photosynthesis varies in relation to the position of the leaf on the tree, which leads to isotopic differences between the cardinal directions. Stable isotopes of tree ring cellulose and other plant material vary in one individual [75, 76] and it is generally suggested to pool several cores/samples in order to get the average representative isotope weight for the tree/year relation [2].

### δ^13^C_*pollen*_ values at different stages of pollen maturation

#### Betula pendula

Mean δ^13^C_pollen_ values of B. pendula do not vary much over the flowering period and both samplings (11 weeks apart) cannot be statistically distinguished (-25.6‰ and -25.7‰; Fig 8, black bar). Pollen primordia are already built by the end of the previous year and the pollen maturation continues after a winter dormancy. In spring, the catkins emerge at the same time as the first leaf buds. Photosynthesis is not yet profitable this early in the year, thus the trees use stored and pooled carbon molecules to build new plant tissue in the beginning of the vegetation period [77]. This explains why the carbon composition of the pollen remained constant.

#### Pinus sylvestris

Mean δ^13^C_*pollen*_ values of *P*. *sylvestris* did not change within 12 days (-26.3‰ and -26.3‰; Fig 8). Comparatively little is known about timing, exact molecular processes and chemical compositions during pollen development [78, 79]. Plants may use only storage molecules to build plant tissue in spring and early summer [13, 77]. However, Loader and Hemming [22] reported a correlation between δ^13^C_*pollen*_ and temperature approximately six weeks prior to pollen release. Thus, at least in parts, *P*. *sylvestris* uses newly accumulated carbon molecules to build pollen. Perhaps we detected no change in the δ^13^C_*pollen*_ of *P*. *sylvestris* because the sporopollenin of the grain wall had already been synthesised during the previous weeks.

### δ^13^C_*pollen*_ values of the elevation transect

δ^13^C_*pollen*_ values of *P*. *abies* increase by 3.4‰ (mean value at 1053 m a.s.l: -26.6‰; mean value at 1344 m a.s.l.: -23.2‰) within the elevation transect covering roughly 300 m at the Tatrzański Mountains. The values correlate with elevation (Adj *R*^*2*^ = 0.52; Fig 9). Although the increase of 3.4‰ is statistically distinct, it cannot be exclusively attributed to an altitudinal effect. In general, the discrimination of δ^13^C is linearly related to the ratio of intercellular to ambient CO_2_ partial pressures, and thus δ^13^C values increase with increasing altitude [80]. Hultine and Marshall [81] found a linear relationship between δ^13^C from *Picea* ssp. needle tissue and elevation where the values of the δ^13^C_*needle*_ increase by approximately 0.5‰ per 300 m in altitude, whereas Warren et al. [82] reported an increase of 2.5‰ over 1000 m of δ^13^C_*wood*_ from 14 different coniferous species. The *P*. *abies* individuals chosen for the elevation transect were all located on the same slope facing north, thus ensuring comparable environmental conditions of e.g. wind, insolation and precipitation. However, the inflorescences from higher elevations were still immature, whereas they were already flowering at lower altitude. Flowering of *P*. *abies* starts with a delay of three days with every additional 100 m of elevation [83]. Bell et al. [15] found that local environmental constraints mask the effect of altitude. They concluded that the source of air masses and moisture input seem to determine the pollen-isotopic weight more than the actual altitude in the mountains. In addition, Treydte et al. [84] pointed out, that an existing temperature signal of *P*. *abies* in δ^13^C_*tree ring*_ was independent of elevation. Influencing factors on pollen-isotope values are very variable in mountainous areas. Which combination of factors caused the high 3.4‰ isotope offset over 300 m remains uninvestigated for now.

### Stepwise regression analysis

#### Factors influencing δ^13^C_*pollen*_ of each species

δ^13^C_*pollen*_ values of all species are determined by several variable factors, and the outcome of the statistical analysis is sometimes inconclusive (Table 7). The factor *site* strongly influences *C*. *avellana* (Prob > F = <0.0001), where the maritime site (Forêt d’Anlier) contrasts the continental sites (Gorczański, Steigerwald, Müritz). *Picea abies* δ^13^C_*pollen*_ values seem to be grouped by altitude, where the mountainous sites of Tre Cime and Tatrzański are contrasting the lower-altitude sites (Forêt d’Anlier, Steigerwald, Gorczański and Liesjärvi). However, these groupings could also be caused by genetic predispositions affecting phenological responses [65, 85, 86]. *Pinus sylvestris* is known to have different haplotypes in Europe, one of which is restricted to the Southern Alps. Another haplotype of *P*. *sylvestris* spreads throughout central Europe [87] and can be found at all other sites of this study. *Pinus sylvestris* has distinct δ^13^C_*pollen*_ values at northern and southern European sites, which seems to be caused by a different reaction to local temperatures due to their genetic background [22].

#### Factors influencing δ^13^C_*pollen*_ at each site

δ^13^C_*pollen*_ values are mostly determined by the factor *species* which largely overprints other local non-climatic factors (e.g. the proximity of the tree to the next water body, type of soil, slope angle; Table 8). Species groupings of the factor *species* at each site are variable and do not seem to follow plant family affiliation or flowering period. In addition, the factor *maturity* is important for the mountainous sites Tre Cime and Tatrański.

### General considerations for the usage of δ^13^C_*pollen*_ in palaeoclimate studies

Species-specific isotope values and patterns display susceptibility to different environmental factors due to their phenology and physiology (Table 3; Figs 4–7). Therefore, the pollen must be separated by species prior to isotope analysis. Without separation, the individual isotope signal within the pollen will be lost and the method cannot be applied in palaeoclimate studies [14, 38]. To date, the separation process is done manually which makes it very time-consuming to pick a sufficient amount of pollen for stable-isotope analysis [38, 88]. The amount of pollen needed to obtain representative average isotopic values is variable. Bell et al. [38] suggest to use a sample size of at least 30 μg carbon when using standard stable-isotope measuring techniques. New technology makes it feasible to decrease this amount. Nelson et al. [35] developed an isotope measuring technique using single pollen grains in order to estimate their photosynthetic pathway and Roij et al. [88] even measured as little as 0.042 μg carbon with a precision better than 0.5‰. However, we identified significantly different pollen-isotope values within one site and even within one tree and therefore suggest using a higher amount of carbon/pollen depending on the pollen type to obtain significant mean pollen-isotope values.

Furthermore, mostly sporopollenin of the pollen wall is preserved in fossil pollen [25]. The analysis of raw pollen material, as reported by this study, shows the general variability between species and sites, but patterns and ranges cannot directly be compared to fossil pollen-isotope values [14]. To accomplish that, extant pollen will have to be treated chemically prior to analysis. The method of sporopollenin-extraction with sulfuric acid has been tested for several species and a consistent offset between raw and the chemically treated pollen material was found [15, 25]. The same accounts for the species of this study: treatment with sulfuric acid resulted in a stable but species-specific offset when compared to raw pollen material (unpublished data).

### δ^18^O_*pollen*_ values of broad-leaved species flowering January to March

#### *Alnus glutinosa* and *Corylus avellana*

Mean δ^18^O_*pollen*_ values of *A*. *glutinosa* (21.1‰) and *C*. *avellana* (21.5‰) of the family Betulaceae are exceptionally low (Table 3). The trees sampled for this study grew on saturated soils with ample water supply out of consistent precipitation throughout the winter months and snowmelt in February and March. Therefore, both species did not face water stress during times of sampling, which may explain the observed low δ^18^O_*pollen*_ values. *Alnus glutinosa* and *C*. *avellana* generally prefer areas with aggravated or missing drainage close to a streamside [89]. In case of insufficient water supply, riparian trees are able to switch sources and especially *A*. *glutinosa* is drought resistant [47]. In general, there is no fractionation of oxygen isotopes during water uptake by the roots [90]. The xylem water remains unaltered during transport until it reaches evaporative tissue or is used to build plant tissue, such as flower primordia and pollen grains [91]. Since *A*. *glutinosa* and *C*. *avellana* flower before leaf proliferation, the water is not passing transpiring leaf tissue, where the isotopes would be altered by evaporative ^18^O enrichment. Mean δ^18^O_*pollen*_ values of *A*. *glutinosa* and *C*. *avellana* from Gorczański are exceptionally low, but display broader ranges than the δ^18^O_*pollen*_ values from Forêt d’Anlier (Fig 4). We expect variable interactions of non-climate environmental influences, e.g. elevation, slope and density of the surrounding vegetation, to have caused the wider distribution of δ^18^O_*pollen*_ values at Gorczański.

### δ^18^O_*pollen*_ of broad-leaved species flowering April to May

#### Acer pseudoplatanus

Mean δ^18^O_*pollen*_ values of *A*. *pseudoplatanus* (Sapindaceae) are highly variable between the sites (range of 18.0‰ to 25.7‰). Mean isotope values are comparable to *B*. *pendula* and *F*. *sylvatica*, depending on the location and year of sampling (Table 3). Noteworthy are the pollen-isotope values at Gorczański, where *A*. *pseudoplatanus* exhibits the lowest δ^18^O_*pollen*_ values of all samplings in this study (2015: 18.0‰, 2016: 18.5‰). Although *A*. *pseudoplatanus* is flowering in May after leaf proliferation, the values are even lower than δ^18^O_*pollen*_ of *A*. *glutinosa* and *C*. *avellana*.

#### *Fagus sylvatica* and *Quercus robur*

δ^18^O_*pollen*_ of *Q*. *robur* are on average 3‰ higher than the values of *F*. *sylvatica* at those sites where both species from the family Fagaceae occur (Forêt d’Anlier and Steigerwald). This likely results from the developmental delay in spring. *Quercus robur* is slower in leaf unfolding, water uptake and in establishing a positive net productivity out of photosynthesis [58, 62]. Oxygen isotope values of precipitation are enriched by evaporation before seeping into the soil, especially during summer months. An increased activity of *Q*. *robur* in summer and the uptake of water enriched in ^18^O might explain the differences in δ^18^O_*pollen*_ between the two species. The δ^18^O_*pollen*_ values of *F*. *sylvatica* from Gorczański are exceptionally low (2015: 18.6‰; 2016: 19.5‰). This was also recognized in the May-flowering species *A*. *pseudoplatanus* (Table 3). However, we do not have a valid explanation for this site-specific phenomenon at the moment.

#### Betula pendula and Carpinus betulus

Mean δ^18^O_*pollen*_ values of species of the Betuloideae deviate by more than 3‰ where *C*. *betulus* (27.1‰) yields higher values than *B*. *pendula* (24.1‰). In general, the mean δ^18^O_*pollen*_ values within the species are very similar between the investigated sites and do not deviate much from the overall mean (Fig 5, dotted line). In particular the 2016 oxygen isotope values of *B*. *pendula* are very similar (Fig 5, black bar), implying that the δ^18^O_*pollen*_ values of these two species are less affected by local environmental events but rather by trans-regional trends. Isotope values of other species deviate more between sites per year (Figs 4, 6 and 7).

### δ^18^O_*pollen*_ values of coniferous trees flowering May to June

#### *Picea abies* and *Pinus sylvestris*

Mean δ^18^O_*pollen*_ values of *P*. *abies* and *P*. *sylvestris* (both Pinaceae) are significantly different, even within the same site (*p(sm)*; Fig 7). Schwarz [13] pointed out that oxygen pollen-isotopes are highly sensitive to environmental conditions. We expect the varying interaction of all local conditions (e.g. micro-climate at the sampling site and water availability) to have caused the different δ^18^O_*pollen*_ values between the vegetation periods 2015 and 2016. However, all European sites compared, both species show similar pollen-isotope patterns. The year-to-year differences between mean δ^18^O_*pollen*_ values of *P*. *abies* from all sites is 1.7‰ (2015: 24.0‰; 2016: 22.3‰), whereas the differences between *P*. *sylvestris* pollen-isotopes is 1.4‰ (2015: 26.8‰; 2016: 25.4‰). With an offset of only 0.3‰ between both, the species seem to react to regional environmental conditions in a similar fashion. Nevertheless, their species-specific isotope values are still very distinct. *Picea abies* (overall average: 23.2‰) and *P*. *sylvestris* (overall average: 26.1‰) yield an average offset of 2.9‰

The δ^18^O_*pollen*_ values at Forêt d’Anlier are higher for 2016 compared to 2015, whereas the δ^18^O_*pollen*_ values at all other locations in this study are higher for 2015 (Fig 7). Both species display the same patterns, therefore weather conditions, which were not investigated further in this study, are expected to have caused the δ^18^O_*pollen*_ inversions. The North Atlantic Oscillation (NAO) is known to influence phenology and temporal variability of seasonal tree development in Europe [92]. The site of Forêt d’Anlier lies closest to the Atlantic Ocean and is therefore more strongly influenced by the NAO than more inland-located sites.

In general, δ^18^O values of precipitation become more negative with increasing altitude and latitude. The δ^18^O_*cellulose*_ of coniferous trees shows a clear latitudinal pattern at a transect in North America covering the area between 65° N and 33° N [93]. Our study covers an area from 60° N to 46° N, but the pollen-isotopes do not show any pattern resembling this latitudinal effect. The northernmost site (Liesjärvi) yields low δ^18^O_*pollen*_ values for *P*. *sylvestris* (23.6‰), but average values of δ^18^O_*pollen*_ for *P*. *abies* (22.7‰). The southernmost site (Tre Cime, 1067m a.s.l.) should yield the highest δ^18^O_*pollen*_ values. However, compared to other central European sites the δ^18^O_*pollen*_ values at Tre Cime are overprinted by an additional altitudinal effect leading to comparatively low δ^18^O_*pollen*_ values for both species (*P*. *abies*: 22.6‰, *P*. *sylvestris*: 24.8‰; Fig 7). All sites of this study are influenced by a variety of location-dependent factors, such as longitude, proximity to the ocean and altitude that overprint the latitudinal effect.

### Intra-tree variability of δ^18^O_*pollen*_

δ^18^O_*pollen*_ values differ between 0.6‰ and 2.1‰ between different cardinal directions in a single tree. Increasing tree height from 1 m to 7 m accounts for a statistically distinct increase in δ^18^O_*pollen*_ of 1.3‰ (Table 4). In general, lower samples yield lower isotope values than upper samples. In addition, the southern and western samples yield lower isotope values than the northern samples (Table 4). The δ^18^O_*pollen*_ intra-branch variability can be as high as 1.4‰ (Table 6), but the average difference between neighbouring inflorescences is 0.5‰, which can be considered to lie within a natural tolerance range.

### δ^18^O_*pollen*_ values at different stages of pollen maturation

#### Betula pendula

δ^18^O_*pollen*_ values (20.9‰ and 24.9‰; Fig 8) are dependent on floral development. Oxygen molecules within the pollen tissue are exchanged until pollination. Rowley et al. [94] reported pollen alteration on the exact day of pollen shedding, where last cells from the pollen developmental tissue are removed and a nutritional transfer takes place. The coating of the outer grain wall contains mostly lipids and proteins and the inside holds cell organelles which develop only late in the pollen maturation process. Therefore, we assume that the variability of the δ^18^O_*pollen*_ values is caused by alteration and exchange of volatile components of the outer and inner pollen grains. Another reason for the highly variable δ^18^O_*pollen*_ of both samplings might be oxidation of the grain wall. The catkins of *B*. *pendula* on 10 March were very young and tightly closed. The ones sampled on 5 May were mostly open, dry and some catkins already exceeded the time of pollination. Therefore, pollen in open catkins may have been oxidized subsequent to air exposure changing the δ^18^O_*pollen*_ values.

#### Pinus sylvestris

Mean δ^18^O_*pollen*_ noticeably changes (25.7‰ and 26.9‰; Fig 8, black arrow) over a time period of 12 days. On 20 May the *P*. *sylvestris* inflorescences were still closed, whereas on 1 June the flowers were open and pollinating. Thus, as with *B*. *pendula*, the difference of δ^18^O_*pollen*_ might result from changes of attached nutrients and volatile grain wall components. In addition, the immature sacculae of coniferous pollen contain a liquid that is only reabsorbed a few days before pollination [94]. The isotope composition of that liquid remains unknown.

### δ^18^O_*pollen*_ values of the elevation transect

δ^18^O_*pollen*_ values do not correlate with altitude (Adj. *R*^*2*^ = 0.14; Fig 9). The mean δ^18^O_*pollen*_ value increases by 1.2‰ over 300 m elevation. However, considering the different maturity of the inflorescences at the time of sampling, the isotopic weight of immature pollen grains on the mountaintop was most likely affected by further maturation.

### Stepwise regression analysis

#### Factors influencing δ^18^O_*pollen*_ of each species

δ^18^O_*pollen*_ values are especially dependent on the geographic location, hence the factor *site* (a combination of latitude, longitude and altitude) particularly important for all species. The broad-leaved trees *A*. *glutinosa* and *C*. *avellana* as well as *F*. *sylvatica* mostly differ between the western sites (Forêt d’Anlier, Steigerwald and Müritz) in relation to the eastern site of Gorczański. The sampling sites for the coniferous pollen are separated in a similar way compared to the broad-leaved species, apart from Gorczański, which is also grouped with the high-latitude site of Liesjärvi and the mountainous site of Tre Cime (Table 7). These groupings indicate differences between maritime and continental (and high-altitude) sampling sites. The factor *maturity* makes a noticeable difference for *A*. *glutinosa*, *F*. *sylvatica* and *P*. *sylvestris* (Table 7), because volatile components and cell organelles increase in size and volume until the end of the pollen maturation process.

#### Factors influencing δ^18^O_*pollen*_ at each site

δ^18^O_*pollen*_ values are also often determined by *species*. However, the statistical groupings are even more variable than those of δ^13^C_*pollen*_. At most of the sites where pollen of the same species were sampled in two consecutive years (Forêt d’Anlier, Tre Cime, Steigerwald and Tatrańzki), the factor *year* significantly affects the pollen-isotope values, reflecting comparative results of Figs 4 to 7 (Table 8).

## Conclusions

1. Most of the δ^13^C_*pollen*_ and δ^18^O_*pollen*_ are normally distributed and all examined species yield specific pollen-isotope patterns, even if the isotope ranges of the species partly overlap.
2. δ^13^C_*pollen*_ and δ^18^O_*pollen*_ ranges show gradients between maritime and continental study sites (W–E transect) as well as between those which differ in day length (N–S transect).
3. Mean species-specific pollen-isotope values vary inter-annually, on average 1.0‰ for δ^13^C_*pollen*_ and 1.6‰ for δ^18^O_*pollen*_. Due to storage of carbon molecules for building new plant tissue, the variation within δ^13^C_*pollen*_ is lower than the variation within δ^18^O_*pollen*_.

1. Broad-leaved tree species flowering before leaf proliferation (January to March; *Alnus glutinosa* and *Corylus avellana*) yield significantly lower δ^13^C_*pollen*_ and δ^18^O_*pollen*_ values than broad-leaved species flowering later in spring (April to May).
2. The coniferous species *Pinus sylvestris* and *Picea abies* show similar reactions to local environmental conditions, but their specific δ^13^C_*pollen*_ and δ^18^O_*pollen*_ values are significantly different.
3. Intra-annual pollen-isotope analysis reveals that δ^13^C_*pollen*_ values do not significantly change during the last stages of the pollen maturation process, whereas δ^18^O_*pollen*_ can be altered during that time.
4. δ^13^C_*pollen*_ and δ^18^O_*pollen*_ differ between sampling positions on trees. Samples from lower positions yield lower isotopic weights than samples from upper positions. Isotope values of single inflorescences can vary within branches up to 1.2‰ for δ^13^C_*pollen*_ and 1.4‰ for δ^18^O_*pollen*_. There is also a circumferential variability of pollen-isotopes at different cardinal directions of up to 3.5‰ for δ^13^C_*pollen*_ and 2.1‰ for δ^18^O_*pollen*_. Because of that variability, an appropriate amount of pollen is needed for stable isotope analysis to enhance the precision of the measured mean δ^13^C_*pollen*_ and δ^18^O_*pollen*_ values.
5. δ^13^C_*pollen*_ values increase with elevation by 3.4‰ over 300 m. Even though the two variates are significantly correlated, the difference is too high to be explained by altitude alone. Thus, additional unknown factors must have influenced the isotopic trend. δ^18^O_*pollen*_ values do not change linearly with elevation.
6. Stepwise regression analysis reveals that the most important factor determining pollen-isotope values of any species in this study is their geographic location (factor *site*). In addition, the statistical analysis shows that the pollen-isotopes within one site are mostly determined by the factor *species*.

Stable carbon and oxygen isotope analysis of fossil pollen can improve the precision of palaeoenvironmental investigations. The applicability of different pollen types in palaeoclimate research relies on the specific plant physiological traits as well as on the pollen abundance in fossil archives. However, due to species-specific pollen-isotope ranges and patterns, fossil pollen should be separated by species as thoroughly as possible. After separation, varying vegetation periods and ecological preferences of the host-plant will allow climate reconstructions on an intra-seasonal scale.

## Supporting information

S1 Dataset Detailed results of the stepwise regression analysis: By species. Eight variables influencing the stable isotope composition in pollen (δ^13^C, δ^18^O) for each species were explored by means of stepwise regression analysis. They include the categorical variables *year* (of sampling), *month* (of sampling), *maturity* (of the pollen at the time of sampling), *slope*, *water* (proximity to water body), *water classification* (type of water body), *soil* and *site*.
(DOCX)

S2 Dataset Detailed results of the stepwise regression analysis: By site. Nine variables influencing the stable isotope composition in pollen (δ^13^C, δ^18^O) at each site were explored by means of stepwise regression analysis. They include the continuous variable *altitude* and the categorical variables *year* (of sampling), *month* (of sampling), *maturity* (of the pollen at the time of sampling), *slope*, *water* (proximity to water body), *water classification* (type of water body), *soil* and *species*.
(DOCX)
