# Supplementary material for: Inter- and intra-tree variability of carbon and oxygen stable isotope ratios of modern pollen from nine European tree species
Source: PLoS One. 2020 Jun 9;15(6):e0234315. doi: 10.1371/journal.pone.0234315 (PMC7282652; doi:10.1371/journal.pone.0234315)
Supplement: S1 Dataset — Eight variables influencing the stable isotope composition in pollen (δ13C, δ18O) for each species were explored by means of stepwise regression analysis. They include the categorical variables year (of sampling), month (of sampling), maturity (of the pollen at the time of sampling), slope, water (proximity to water body), water classification (type of water body), soil and site. (DOCX) [file pone.0234315.s001.docx]

**Detailed results of the Stepwise Regression Analysis: Modelling by Species**

**Species: *Acer pseudoplatanus***

**Environmental factors affecting δ^13^C**

**Response d13c**

**Whole Model**

**Regression Plot**

**Actual by Predicted Plot**

**Residual by Predicted Plot**

**Summary of Fit**

| RSquare | 0,148744 |
| --- | --- |
| RSquare Adj | 0,121284 |
| Root Mean Square Error | 1,376285 |
| Mean of Response | -25,1081 |
| Observations (or Sum Wgts) | 33 |

**Analysis of Variance**

| **Source** | **DF** | **Sum of Squares** | **Mean Square** | **F Ratio** |
| --- | --- | --- | --- | --- |
| Model | 1 | 10,260226 | 10,2602 | 5,4168 |
| Error | 31 | 58,718945 | 1,8942 | **Prob > F** |
| C. Total | 32 | 68,979170 |  | 0,0266* |

**Parameter Estimates**

| **Term** | **Estimate** | **Std Error** | **t Ratio** | **Prob>\|t\|** | **VIF** |
| --- | --- | --- | --- | --- | --- |
| Intercept | -26,13488 | 0,502041 | -52,06 | <,0001* | . |
| water{2-1&0} | -1,168449 | 0,502041 | -2,33 | 0,0266* | 1 |

**water{2-1&0}**

**Leverage Plot**

**Environmental factors affecting δ^18^O**

**Response d18o**

**Whole Model**

**Actual by Predicted Plot**

**Effect Summary**

| **Source** | **LogWorth** |  | **PValue** |
| --- | --- | --- | --- |
| site{GOR-DOL&STE&ARD} | 16,992 |  | 0,00000 |
| site{DOL&STE-ARD} | 2,366 |  | 0,00431 |

**Residual by Predicted Plot**

**Summary of Fit**

| RSquare | 0,916443 |
| --- | --- |
| RSquare Adj | 0,910873 |
| Root Mean Square Error | 1,016799 |
| Mean of Response | 21,37054 |
| Observations (or Sum Wgts) | 33 |

**Analysis of Variance**

| **Source** | **DF** | **Sum of Squares** | **Mean Square** | **F Ratio** |
| --- | --- | --- | --- | --- |
| Model | 2 | 340,18545 | 170,093 | 164,5187 |
| Error | 30 | 31,01643 | 1,034 | **Prob > F** |
| C. Total | 32 | 371,20188 |  | <,0001* |

**Parameter Estimates**

| **Term** | **Estimate** | **Std Error** | **t Ratio** | **Prob>\|t\|** | **VIF** |
| --- | --- | --- | --- | --- | --- |
| Intercept | 21,567812 | 0,180099 | 119,76 | <,0001* | . |
| site{GOR-DOL&STE&ARD} | -3,266636 | 0,180099 | -18,14 | <,0001* | 1,0343434 |
| site{DOL&STE-ARD} | -0,810829 | 0,262536 | -3,09 | 0,0043* | 1,0343434 |

**site{GOR-DOL&STE&ARD}**

**Leverage Plot**

**site{DOL&STE-ARD}**

**Leverage Plot**

**Species: *Alnus glutinosa***

**Environmental factors affecting δ^13^C**

**Response d13c**

**Whole Model**

**Actual by Predicted Plot**

**Effect Summary**

| **Source** | **LogWorth** |  | **PValue** |
| --- | --- | --- | --- |
| maturity{0&-1-1} | 2,923 |  | 0,00119 |
| soil1{HL&DC-HL-DC} | 2,012 |  | 0,00972 |
| soil1{HL-DC} | 1,853 |  | 0,01404 |
| site{MUR&ARD-GOR&STE} | 0,472 |  | 0,33701 |

**Residual by Predicted Plot**

**Summary of Fit**

| RSquare | 0,416046 |
| --- | --- |
| RSquare Adj | 0,388885 |
| Root Mean Square Error | 1,643958 |
| Mean of Response | -28,0828 |
| Observations (or Sum Wgts) | 91 |

**Analysis of Variance**

| **Source** | **DF** | **Sum of Squares** | **Mean Square** | **F Ratio** |
| --- | --- | --- | --- | --- |
| Model | 4 | 165,59326 | 41,3983 | 15,3180 |
| Error | 86 | 232,42352 | 2,7026 | **Prob > F** |
| C. Total | 90 | 398,01678 |  | <,0001* |

**Parameter Estimates**

| **Term** | **Estimate** | **Std Error** | **t Ratio** | **Prob>\|t\|** | **VIF** |
| --- | --- | --- | --- | --- | --- |
| Intercept | -26,55668 | 0,612966 | -43,32 | <,0001* | . |
| site{MUR&ARD-GOR&STE} | 0,8127337 | 0,841789 | 0,97 | 0,3370 | 23,856875 |
| maturity{0&-1-1} | -1,995418 | 0,595235 | -3,35 | 0,0012* | 2,0053605 |
| soil1{HL&DC-HL-DC} | -2,233991 | 0,84472 | -2,64 | 0,0097* | 23,953685 |
| soil1{HL-DC} | -0,976316 | 0,38937 | -2,51 | 0,0140* | 1,8025194 |

**site{MUR&ARD-GOR&STE}**

**Leverage Plot**

**maturity{0&-1-1}**

**Leverage Plot**

**soil1{HL&DC-HL-DC}**

**Leverage Plot**

**soil1{HL-DC}**

**Leverage Plot**

**Environmental factors affecting δ^18^O**

**Response d18o**

**Whole Model**

**Actual by Predicted Plot**

**Effect Summary**

| **Source** | **LogWorth** |  | **PValue** |
| --- | --- | --- | --- |
| site{GOR-ARD&MUR&STE} | 26,549 |  | 0,00000 |
| maturity{-1&0-1} | 7,438 |  | 0,00000 |
| site{MUR-STE} | 3,040 |  | 0,00091 |
| year | 2,466 |  | 0,00342 |
| site{ARD-MUR&STE} | 1,792 |  | 0,01615 |

**Lack Of Fit**

| **Source** | **DF** | **Sum of Squares** | **Mean Square** | **F Ratio** |
| --- | --- | --- | --- | --- |
| Lack Of Fit | 1 | 3,235725 | 3,23572 | 9,1385 |
| Pure Error | 84 | 29,742256 | 0,35407 | **Prob > F** |
| Total Error | 85 | 32,977981 |  | 0,0033* |
|  |  |  |  | **Max RSq** |
|  |  |  |  | 0,9112 |

**Residual by Predicted Plot**

**Summary of Fit**

| RSquare | 0,901548 |
| --- | --- |
| RSquare Adj | 0,895756 |
| Root Mean Square Error | 0,622877 |
| Mean of Response | 20,3981 |
| Observations (or Sum Wgts) | 91 |

**Analysis of Variance**

| **Source** | **DF** | **Sum of Squares** | **Mean Square** | **F Ratio** |
| --- | --- | --- | --- | --- |
| Model | 5 | 301,98540 | 60,3971 | 155,6721 |
| Error | 85 | 32,97798 | 0,3880 | **Prob > F** |
| C. Total | 90 | 334,96338 |  | <,0001* |

**Parameter Estimates**

| **Term** | **Estimate** | **Std Error** | **t Ratio** | **Prob>\|t\|** | **VIF** |
| --- | --- | --- | --- | --- | --- |
| Intercept | 21,362281 | 0,184583 | 115,73 | <,0001* | . |
| year[2015] | -0,207969 | 0,069053 | -3,01 | 0,0034* | 1,1150289 |
| site{GOR-ARD&MUR&STE} | -1,541418 | 0,096674 | -15,94 | <,0001* | 2,1854416 |
| site{ARD-MUR&STE} | 0,4408347 | 0,179612 | 2,45 | 0,0162* | 2,9349275 |
| site{MUR-STE} | 1,1920018 | 0,346784 | 3,44 | 0,0009* | 2,1391005 |
| maturity{-1&0-1} | -1,386169 | 0,22883 | -6,06 | <,0001* | 2,0645226 |

**year**

**Leverage Plot**

**Least Squares Means Table**

| **Level** | **Least Sq Mean** | **Std Error** | **Mean** |
| --- | --- | --- | --- |
| 2015 | 20,178702 | 0,09782715 | 19,8656 |
| 2016 | 20,594640 | 0,09231563 | 20,8751 |

**site{GOR-ARD&MUR&STE}**

**Leverage Plot**

**site{ARD-MUR&STE}**

**Leverage Plot**

**site{MUR-STE}**

**Leverage Plot**

**maturity{-1&0-1}**

**Leverage Plot**

**Species: *Betula pendula***

**Environmental factors affecting δ^13^C**

**Response d13c**

**Whole Model**

**Actual by Predicted Plot**

**Residual by Predicted Plot**

**Summary of Fit**

| RSquare | 0,322129 |
| --- | --- |
| RSquare Adj | 0,312305 |
| Root Mean Square Error | 1,358632 |
| Mean of Response | -24,6769 |
| Observations (or Sum Wgts) | 71 |

**Analysis of Variance**

| **Source** | **DF** | **Sum of Squares** | **Mean Square** | **F Ratio** |
| --- | --- | --- | --- | --- |
| Model | 1 | 60,52516 | 60,5252 | 32,7893 |
| Error | 69 | 127,36576 | 1,8459 | **Prob > F** |
| C. Total | 70 | 187,89093 |  | <,0001* |

**Parameter Estimates**

| **Term** | **Estimate** | **Std Error** | **t Ratio** | **Prob>\|t\|** | **VIF** |
| --- | --- | --- | --- | --- | --- |
| Intercept | -24,74204 | 0,161641 | -153,1 | <,0001* | . |
| year[2015] | -0,925589 | 0,161641 | -5,73 | <,0001* | 1 |

**year**

**Leverage Plot**

**Least Squares Means Table**

| **Level** | **Least Sq Mean** | **Std Error** | **Mean** |
| --- | --- | --- | --- |
| 2015 | -25,66763 | 0,23650745 | -25,668 |
| 2016 | -23,81645 | 0,22039920 | -23,816 |

**Environmental factors affecting δ^18^O**

**Response d18o**

**Whole Model**

**Regression Plot**

**Actual by Predicted Plot**

**Effect Summary**

| **Source** | **LogWorth** |  | **PValue** |
| --- | --- | --- | --- |
| month{mar-apr&may} | 17,308 |  | 0,00000 |
| year | 1,783 |  | 0,01649 |

**Residual by Predicted Plot**

**Summary of Fit**

| RSquare | 0,686792 |
| --- | --- |
| RSquare Adj | 0,67758 |
| Root Mean Square Error | 0,906048 |
| Mean of Response | 23,92133 |
| Observations (or Sum Wgts) | 71 |

**Analysis of Variance**

| **Source** | **DF** | **Sum of Squares** | **Mean Square** | **F Ratio** |
| --- | --- | --- | --- | --- |
| Model | 2 | 122,40626 | 61,2031 | 74,5540 |
| Error | 68 | 55,82282 | 0,8209 | **Prob > F** |
| C. Total | 70 | 178,22907 |  | <,0001* |

**Parameter Estimates**

| **Term** | **Estimate** | **Std Error** | **t Ratio** | **Prob>\|t\|** | **VIF** |
| --- | --- | --- | --- | --- | --- |
| Intercept | 22,585381 | 0,155107 | 145,61 | <,0001* | . |
| year[2015] | 0,2984064 | 0,121365 | 2,46 | 0,0165* | 1,2676056 |
| month{mar-apr&may} | -1,966212 | 0,16729 | -11,75 | <,0001* | 1,2676056 |

**year**

**Leverage Plot**

**Least Squares Means Table**

| **Level** | **Least Sq Mean** | **Std Error** | **Mean** |
| --- | --- | --- | --- |
| 2015 | 24,240751 | 0,16863993 | 23,5392 |
| 2016 | 23,643938 | 0,15585337 | 24,2532 |

**month{mar-apr&may}**

**Leverage Plot**

**Species: *Carpinus betulus***

**Environmental factors affecting δ^13^C**

**Response d13c**

**Whole Model**

**Regression Plot**

**Actual by Predicted Plot**

**Residual by Predicted Plot**

**Summary of Fit**

| RSquare | 0,2847 |
| --- | --- |
| RSquare Adj | 0,242623 |
| Root Mean Square Error | 1,499275 |
| Mean of Response | -25,7759 |
| Observations (or Sum Wgts) | 19 |

**Analysis of Variance**

| **Source** | **DF** | **Sum of Squares** | **Mean Square** | **F Ratio** |
| --- | --- | --- | --- | --- |
| Model | 1 | 15,209342 | 15,2093 | 6,7662 |
| Error | 17 | 38,213016 | 2,2478 | **Prob > F** |
| C. Total | 18 | 53,422358 |  | 0,0186* |

**Parameter Estimates**

| **Term** | **Estimate** | **Std Error** | **t Ratio** | **Prob>\|t\|** | **VIF** |
| --- | --- | --- | --- | --- | --- |
| Intercept | -26,2571 | 0,390552 | -67,23 | <,0001* | . |
| water_classification{1-0&2} | -1,015905 | 0,390552 | -2,60 | 0,0186* | 1 |

**water_classification{1-0&2}**

**Leverage Plot**

**Environmental factors affecting δ^18^O**

**Response d18o**

**Whole Model**

**Actual by Predicted Plot**

**Effect Summary**

| **Source** | **LogWorth** |  | **PValue** |
| --- | --- | --- | --- |
| water{1&0-2} | 2,323 |  | 0,00475 |
| maturity{1&0--1} | 1,020 |  | 0,09546 |

**Residual by Predicted Plot**

**Summary of Fit**

| RSquare | 0,448635 |
| --- | --- |
| RSquare Adj | 0,379714 |
| Root Mean Square Error | 0,788789 |
| Mean of Response | 26,30588 |
| Observations (or Sum Wgts) | 19 |

**Analysis of Variance**

| **Source** | **DF** | **Sum of Squares** | **Mean Square** | **F Ratio** |
| --- | --- | --- | --- | --- |
| Model | 2 | 8,100207 | 4,05010 | 6,5094 |
| Error | 16 | 9,955017 | 0,62219 | **Prob > F** |
| C. Total | 18 | 18,055224 |  | 0,0085* |

**Parameter Estimates**

| **Term** | **Estimate** | **Std Error** | **t Ratio** | **Prob>\|t\|** | **VIF** |
| --- | --- | --- | --- | --- | --- |
| Intercept | 27,715417 | 0,483033 | 57,38 | <,0001* | . |
| maturity{1&0--1} | -0,720313 | 0,406533 | -1,77 | 0,0955 | 1,0065789 |
| water{1&0-2} | -0,969062 | 0,295796 | -3,28 | 0,0048* | 1,0065789 |

**maturity{1&0--1}**

**Leverage Plot**

**water{1&0-2}**

**Leverage Plot**

**Species: *Corylus avellana***

**Environmental factors affecting δ^13^C**

**Response d13c**

**Whole Model**

**Actual by Predicted Plot**

**Effect Summary**

| **Source** | **LogWorth** |  | **PValue** |
| --- | --- | --- | --- |
| site{ARD-GOR&STE&MUR} | 8,433 |  | 0,00000 |
| year | 2,327 |  | 0,00471 |
| site{GOR&STE-MUR} | 1,846 |  | 0,01427 |

**Lack Of Fit**

| **Source** | **DF** | **Sum of Squares** | **Mean Square** | **F Ratio** |
| --- | --- | --- | --- | --- |
| Lack Of Fit | 1 | 2,74630 | 2,74630 | 1,5892 |
| Pure Error | 87 | 150,34464 | 1,72810 | **Prob > F** |
| Total Error | 88 | 153,09094 |  | 0,2108 |
|  |  |  |  | **Max RSq** |
|  |  |  |  | 0,4302 |

**Residual by Predicted Plot**

**Summary of Fit**

| RSquare | 0,419824 |
| --- | --- |
| RSquare Adj | 0,400046 |
| Root Mean Square Error | 1,318965 |
| Mean of Response | -27,7823 |
| Observations (or Sum Wgts) | 92 |

**Analysis of Variance**

| **Source** | **DF** | **Sum of Squares** | **Mean Square** | **F Ratio** |
| --- | --- | --- | --- | --- |
| Model | 3 | 110,77905 | 36,9263 | 21,2261 |
| Error | 88 | 153,09094 | 1,7397 | **Prob > F** |
| C. Total | 91 | 263,86999 |  | <,0001* |

**Parameter Estimates**

| **Term** | **Estimate** | **Std Error** | **t Ratio** | **Prob>\|t\|** | **VIF** |
| --- | --- | --- | --- | --- | --- |
| Intercept | -27,60614 | 0,179477 | -153,8 | <,0001* | . |
| year[2015] | -0,412473 | 0,142228 | -2,90 | 0,0047* | 1,0652174 |
| site{ARD-GOR&STE&MUR} | -1,176091 | 0,179477 | -6,55 | <,0001* | 1,6906138 |
| site{GOR&STE-MUR} | -0,739247 | 0,295682 | -2,50 | 0,0143* | 1,7239808 |

**year**

**Leverage Plot**

**Least Squares Means Table**

| **Level** | **Least Sq Mean** | **Std Error** | **Mean** |
| --- | --- | --- | --- |
| 2015 | -28,22169 | 0,20460386 | -28,369 |
| 2016 | -27,39674 | 0,19127384 | -27,267 |

**site{ARD-GOR&STE&MUR}**

**Leverage Plot**

**site{GOR&STE-MUR}**

**Leverage Plot**

**Environmental factors affecting δ^18^O**

**Response d18o**

**Whole Model**

**Actual by Predicted Plot**

**Effect Summary**

| **Source** | **LogWorth** |  | **PValue** |
| --- | --- | --- | --- |
| site{GOR-ARD&STE&MUR} | 29,356 |  | 0,00000 |
| site{ARD-STE&MUR} | 3,567 |  | 0,00027 |

**Residual by Predicted Plot**

**Summary of Fit**

| RSquare | 0,790852 |
| --- | --- |
| RSquare Adj | 0,786152 |
| Root Mean Square Error | 1,018627 |
| Mean of Response | 20,89025 |
| Observations (or Sum Wgts) | 92 |

**Analysis of Variance**

| **Source** | **DF** | **Sum of Squares** | **Mean Square** | **F Ratio** |
| --- | --- | --- | --- | --- |
| Model | 2 | 349,18941 | 174,595 | 168,2676 |
| Error | 89 | 92,34654 | 1,038 | **Prob > F** |
| C. Total | 91 | 441,53596 |  | <,0001* |

**Parameter Estimates**

| **Term** | **Estimate** | **Std Error** | **t Ratio** | **Prob>\|t\|** | **VIF** |
| --- | --- | --- | --- | --- | --- |
| Intercept | 20,977239 | 0,125803 | 166,75 | <,0001* | . |
| site{GOR-ARD&STE&MUR} | -2,166733 | 0,125803 | -17,22 | <,0001* | 1,392663 |
| site{ARD-STE&MUR} | -0,745195 | 0,196472 | -3,79 | 0,0003* | 1,392663 |

**site{GOR-ARD&STE&MUR}**

**Leverage Plot**

**site{ARD-STE&MUR}**

**Leverage Plot**

**Species: *Fagus sylvatica***

**Environmental factors affecting δ^13^C**

**Response d13c**

**Whole Model**

**Regression Plot**

**Actual by Predicted Plot**

**Residual by Predicted Plot**

**Summary of Fit**

| RSquare | 0,241421 |
| --- | --- |
| RSquare Adj | 0,227628 |
| Root Mean Square Error | 1,364345 |
| Mean of Response | -27,1091 |
| Observations (or Sum Wgts) | 57 |

**Analysis of Variance**

| **Source** | **DF** | **Sum of Squares** | **Mean Square** | **F Ratio** |
| --- | --- | --- | --- | --- |
| Model | 1 | 32,58251 | 32,5825 | 17,5039 |
| Error | 55 | 102,37911 | 1,8614 | **Prob > F** |
| C. Total | 56 | 134,96161 |  | 0,0001* |

**Parameter Estimates**

| **Term** | **Estimate** | **Std Error** | **t Ratio** | **Prob>\|t\|** | **VIF** |
| --- | --- | --- | --- | --- | --- |
| Intercept | -26,46392 | 0,237565 | -111,4 | <,0001* | . |
| site{GOR&DOL&ARD-STE} | -0,993918 | 0,237565 | -4,18 | 0,0001* | 1 |

**site{GOR&DOL&ARD-STE}**

**Leverage Plot**

**Environmental factors affecting δ^18^O**

**Response d18o**

**Whole Model**

**Actual by Predicted Plot**

**Effect Summary**

| **Source** | **LogWorth** |  | **PValue** |
| --- | --- | --- | --- |
| site{GOR-ARD&STE&DOL} | 24,757 |  | 0,00000 |
| maturity{0-1} | 4,389 |  | 0,00004 |
| maturity{0&1--1} | 0,165 |  | 0,68435 |

**Lack Of Fit**

| **Source** | **DF** | **Sum of Squares** | **Mean Square** | **F Ratio** |
| --- | --- | --- | --- | --- |
| Lack Of Fit | 1 | 0,673157 | 0,673157 | 0,9960 |
| Pure Error | 52 | 35,146272 | 0,675890 | **Prob > F** |
| Total Error | 53 | 35,819428 |  | 0,3229 |
|  |  |  |  | **Max RSq** |
|  |  |  |  | 0,8834 |

**Residual by Predicted Plot**

**Summary of Fit**

| RSquare | 0,881141 |
| --- | --- |
| RSquare Adj | 0,874413 |
| Root Mean Square Error | 0,822094 |
| Mean of Response | 21,66615 |
| Observations (or Sum Wgts) | 57 |

**Analysis of Variance**

| **Source** | **DF** | **Sum of Squares** | **Mean Square** | **F Ratio** |
| --- | --- | --- | --- | --- |
| Model | 3 | 265,54005 | 88,5134 | 130,9682 |
| Error | 53 | 35,81943 | 0,6758 | **Prob > F** |
| C. Total | 56 | 301,35948 |  | <,0001* |

**Parameter Estimates**

| **Term** | **Estimate** | **Std Error** | **t Ratio** | **Prob>\|t\|** | **VIF** |
| --- | --- | --- | --- | --- | --- |
| Intercept | 21,688116 | 0,253851 | 85,44 | <,0001* | . |
| site{GOR-ARD&STE&DOL} | -2,219135 | 0,115836 | -19,16 | <,0001* | 1,0895279 |
| maturity{0&1--1} | -0,10386 | 0,254074 | -0,41 | 0,6843 | 1,0858709 |
| maturity{0-1} | -0,636418 | 0,142219 | -4,47 | <,0001* | 1,078451 |

**site{GOR-ARD&STE&DOL}**

**Leverage Plot**

**maturity{0&1--1}**

**Leverage Plot**

**maturity{0-1}**

**Leverage Plot**

**Species: *Picea abies***

**Environmental factors affecting δ^13^C**

**Response d13c**

**Whole Model**

**Actual by Predicted Plot**

**Effect Summary**

| **Source** | **LogWorth** |  | **PValue** |
| --- | --- | --- | --- |
| site{ARD&STE&GOR&FIN-TAT&DOL} | 12,200 |  | 0,00000 |
| site{TAT-DOL} | 2,652 |  | 0,00223 |

**Residual by Predicted Plot**

**Summary of Fit**

| RSquare | 0,390784 |
| --- | --- |
| RSquare Adj | 0,38119 |
| Root Mean Square Error | 1,163038 |
| Mean of Response | -25,2415 |
| Observations (or Sum Wgts) | 130 |

**Analysis of Variance**

| **Source** | **DF** | **Sum of Squares** | **Mean Square** | **F Ratio** |
| --- | --- | --- | --- | --- |
| Model | 2 | 110,19367 | 55,0968 | 40,7323 |
| Error | 127 | 171,78743 | 1,3527 | **Prob > F** |
| C. Total | 129 | 281,98111 |  | <,0001* |

**Parameter Estimates**

| **Term** | **Estimate** | **Std Error** | **t Ratio** | **Prob>\|t\|** | **VIF** |
| --- | --- | --- | --- | --- | --- |
| Intercept | -24,92509 | 0,111383 | -223,8 | <,0001* | . |
| site{ARD&STE&GOR&FIN-TAT&DOL} | -0,892294 | 0,111383 | -8,01 | <,0001* | 1,0159355 |
| site{TAT-DOL} | -0,580634 | 0,185997 | -3,12 | 0,0022* | 1,0159355 |

**site{ARD&STE&GOR&FIN-TAT&DOL}**

**Leverage Plot**

**site{TAT-DOL}**

**Leverage Plot**

**Environmental factors affecting δ^18^O**

**Response d18o**

**Whole Model**

**Actual by Predicted Plot**

**Effect Summary**

| **Source** | **LogWorth** |  | **PValue** |
| --- | --- | --- | --- |
| year | 11,265 |  | 0,00000 |
| water_classification{0&2-1} | 6,034 |  | 0,00000 |
| site{DOL&TAT&FIN&GOR-STE&ARD} | 4,239 |  | 0,00006 |
| site{DOL&TAT-FIN} | 2,165 |  | 0,00684 |
| site{DOL&TAT&FIN-GOR} | 1,029 |  | 0,09364 |

**Lack Of Fit**

| **Source** | **DF** | **Sum of Squares** | **Mean Square** | **F Ratio** |
| --- | --- | --- | --- | --- |
| Lack Of Fit | 5 | 43,76722 | 8,75344 | 7,8479 |
| Pure Error | 119 | 132,73062 | 1,11538 | **Prob > F** |
| Total Error | 124 | 176,49784 |  | <,0001* |
|  |  |  |  | **Max RSq** |
|  |  |  |  | 0,6414 |

**Residual by Predicted Plot**

**Summary of Fit**

| RSquare | 0,523151 |
| --- | --- |
| RSquare Adj | 0,503923 |
| Root Mean Square Error | 1,193051 |
| Mean of Response | 23,36985 |
| Observations (or Sum Wgts) | 130 |

**Analysis of Variance**

| **Source** | **DF** | **Sum of Squares** | **Mean Square** | **F Ratio** |
| --- | --- | --- | --- | --- |
| Model | 5 | 193,63583 | 38,7272 | 27,2081 |
| Error | 124 | 176,49784 | 1,4234 | **Prob > F** |
| C. Total | 129 | 370,13366 |  | <,0001* |

**Parameter Estimates**

| **Term** | **Estimate** | **Std Error** | **t Ratio** | **Prob>\|t\|** | **VIF** |
| --- | --- | --- | --- | --- | --- |
| Intercept | 24,266672 | 0,152037 | 159,61 | <,0001* | . |
| year[2015] | 0,9183631 | 0,12039 | 7,63 | <,0001* | 1,3209295 |
| site{DOL&TAT&FIN&GOR-STE&ARD} | -0,51527 | 0,123699 | -4,17 | <,0001* | 1,3019352 |
| site{DOL&TAT&FIN-GOR} | -0,256798 | 0,151999 | -1,69 | 0,0936 | 1,2905361 |
| site{DOL&TAT-FIN} | -0,606403 | 0,220457 | -2,75 | 0,0068* | 1,4708741 |
| water_classification{0&2-1} | -0,842186 | 0,163005 | -5,17 | <,0001* | 1,4135519 |

**year**

**Leverage Plot**

**Least Squares Means Table**

| **Level** | **Least Sq Mean** | **Std Error** | **Mean** |
| --- | --- | --- | --- |
| 2015 | 24,330595 | 0,16374212 | 23,9706 |
| 2016 | 22,493869 | 0,15535674 | 22,8221 |

**site{DOL&TAT&FIN&GOR-STE&ARD}**

**Leverage Plot**

**site{DOL&TAT&FIN-GOR}**

**Leverage Plot**

**site{DOL&TAT-FIN}**

**Leverage Plot**

**water_classification{0&2-1}**

**Leverage Plot**

**Species: *Pinus sylvestris***

**Environmental factors affecting δ^13^C**

**Response d13c**

**Whole Model**

**Actual by Predicted Plot**

**Effect Summary**

| **Source** | **LogWorth** |  | **PValue** |
| --- | --- | --- | --- |
| maturity{1&-1-0} | 3,051 |  | 0,00089 |
| soil1{HP&HL-DC-HL&GL&RL-DC&EF&DC} | 2,746 |  | 0,00179 |

**Lack Of Fit**

| **Source** | **DF** | **Sum of Squares** | **Mean Square** | **F Ratio** |
| --- | --- | --- | --- | --- |
| Lack Of Fit | 1 | 0,56441 | 0,56441 | 0,5337 |
| Pure Error | 124 | 131,13081 | 1,05751 | **Prob > F** |
| Total Error | 125 | 131,69523 |  | 0,4664 |
|  |  |  |  | **Max RSq** |
|  |  |  |  | 0,2936 |

**Residual by Predicted Plot**

**Summary of Fit**

| RSquare | 0,290535 |
| --- | --- |
| RSquare Adj | 0,279183 |
| Root Mean Square Error | 1,026432 |
| Mean of Response | -26,7327 |
| Observations (or Sum Wgts) | 128 |

**Analysis of Variance**

| **Source** | **DF** | **Sum of Squares** | **Mean Square** | **F Ratio** |
| --- | --- | --- | --- | --- |
| Model | 2 | 53,93084 | 26,9654 | 25,5945 |
| Error | 125 | 131,69523 | 1,0536 | **Prob > F** |
| C. Total | 127 | 185,62607 |  | <,0001* |

**Parameter Estimates**

| **Term** | **Estimate** | **Std Error** | **t Ratio** | **Prob>\|t\|** | **VIF** |
| --- | --- | --- | --- | --- | --- |
| Intercept | -27,08926 | 0,116942 | -231,6 | <,0001* | . |
| maturity{1&-1-0} | -0,386929 | 0,113637 | -3,40 | 0,0009* | 1,4937937 |
| soil1{HP&HL-DC-HL&GL&RL-DC&EF&DC} | -0,446229 | 0,13985 | -3,19 | 0,0018* | 1,4937937 |

**maturity{1&-1-0}**

**Leverage Plot**

**soil1{HP&HL-DC-HL&GL&RL-DC&EF&DC}**

**Leverage Plot**

**Environmental factors affecting δ^18^O**

**Response d18o**

**Whole Model**

**Actual by Predicted Plot**

**Effect Summary**

| **Source** | **LogWorth** |  | **PValue** |
| --- | --- | --- | --- |
| maturity{1&-1-0} | 11,462 |  | 0,00000 |
| site{FIN&DOL&GOR-STE&ARD&MUR} | 10,843 |  | 0,00000 |
| month | 1,905 |  | 0,01244 |
| soil1{HP&HL-DC&RL-DC-EF&GL&DC&HL} | 0,632 |  | 0,23342 |

**Lack Of Fit**

| **Source** | **DF** | **Sum of Squares** | **Mean Square** | **F Ratio** |
| --- | --- | --- | --- | --- |
| Lack Of Fit | 1 | 0,15257 | 0,15257 | 0,0747 |
| Pure Error | 122 | 249,07840 | 2,04163 | **Prob > F** |
| Total Error | 123 | 249,23097 |  | 0,7850 |
|  |  |  |  | **Max RSq** |
|  |  |  |  | 0,5965 |

**Residual by Predicted Plot**

**Summary of Fit**

| RSquare | 0,596278 |
| --- | --- |
| RSquare Adj | 0,583149 |
| Root Mean Square Error | 1,42347 |
| Mean of Response | 25,89587 |
| Observations (or Sum Wgts) | 128 |

**Analysis of Variance**

| **Source** | **DF** | **Sum of Squares** | **Mean Square** | **F Ratio** |
| --- | --- | --- | --- | --- |
| Model | 4 | 368,10257 | 92,0256 | 45,4163 |
| Error | 123 | 249,23097 | 2,0263 | **Prob > F** |
| C. Total | 127 | 617,33355 |  | <,0001* |

**Parameter Estimates**

| **Term** | **Estimate** | **Std Error** | **t Ratio** | **Prob>\|t\|** | **VIF** |
| --- | --- | --- | --- | --- | --- |
| Intercept | 26,812005 | 0,284445 | 94,26 | <,0001* | . |
| site{FIN&DOL&GOR-STE&ARD&MUR} | -1,592913 | 0,213834 | -7,45 | <,0001* | 2,3356004 |
| month[may] | -0,610587 | 0,240709 | -2,54 | 0,0124* | 1,6861979 |
| maturity{1&-1-0} | -1,176431 | 0,152372 | -7,72 | <,0001* | 1,3964503 |
| soil1{HP&HL-DC&RL-DC-EF&GL&DC&HL} | 0,2576401 | 0,215153 | 1,20 | 0,2334 | 2,8892266 |

**site{FIN&DOL&GOR-STE&ARD&MUR}**

**Leverage Plot**

**month**

**Leverage Plot**

**Least Squares Means Table**

| **Level** | **Least Sq Mean** | **Std Error** | **Mean** |
| --- | --- | --- | --- |
| may | 25,733683 | 0,14113227 | 25,7653 |
| june | 26,954857 | 0,43602642 | 26,7485 |

**maturity{1&-1-0}**

**Leverage Plot**

**soil1{HP&HL-DC&RL-DC-EF&GL&DC&HL}**

**Leverage Plot**

**Species: *Quercus robur***

**Environmental factors affecting δ^13^C**

**Response d13c**

**Whole Model**

**Actual by Predicted Plot**

**Residual by Predicted Plot**

**Summary of Fit**

| RSquare | 0,133711 |
| --- | --- |
| RSquare Adj | 0,109648 |
| Root Mean Square Error | 1,372853 |
| Mean of Response | -25,8094 |
| Observations (or Sum Wgts) | 38 |

**Analysis of Variance**

| **Source** | **DF** | **Sum of Squares** | **Mean Square** | **F Ratio** |
| --- | --- | --- | --- | --- |
| Model | 1 | 10,472652 | 10,4727 | 5,5566 |
| Error | 36 | 67,850155 | 1,8847 | **Prob > F** |
| C. Total | 37 | 78,322807 |  | 0,0240* |

**Parameter Estimates**

| **Term** | **Estimate** | **Std Error** | **t Ratio** | **Prob>\|t\|** | **VIF** |
| --- | --- | --- | --- | --- | --- |
| Intercept | -25,89336 | 0,225535 | -114,8 | <,0001* | . |
| year[2015] | -0,531641 | 0,225535 | -2,36 | 0,0240* | 1 |

**year**

**Leverage Plot**

**Least Squares Means Table**

| **Level** | **Least Sq Mean** | **Std Error** | **Mean** |
| --- | --- | --- | --- |
| 2015 | -26,42500 | 0,34321336 | -26,425 |
| 2016 | -25,36172 | 0,29269333 | -25,362 |

**Environmental factors affecting δ^18^O**

**Response d18o**

**Whole Model**

**Actual by Predicted Plot**

**Residual by Predicted Plot**

**Summary of Fit**

| RSquare | 0,154555 |
| --- | --- |
| RSquare Adj | 0,131071 |
| Root Mean Square Error | 1,214698 |
| Mean of Response | 26,81547 |
| Observations (or Sum Wgts) | 38 |

**Analysis of Variance**

| **Source** | **DF** | **Sum of Squares** | **Mean Square** | **F Ratio** |
| --- | --- | --- | --- | --- |
| Model | 1 | 9,710419 | 9,71042 | 6,5811 |
| Error | 36 | 53,117669 | 1,47549 | **Prob > F** |
| C. Total | 37 | 62,828087 |  | 0,0146* |

**Parameter Estimates**

| **Term** | **Estimate** | **Std Error** | **t Ratio** | **Prob>\|t\|** | **VIF** |
| --- | --- | --- | --- | --- | --- |
| Intercept | 26,734637 | 0,199553 | 133,97 | <,0001* | . |
| year[2015] | -0,511929 | 0,199553 | -2,57 | 0,0146* | 1 |

**year**

**Leverage Plot**

**Least Squares Means Table**

| **Level** | **Least Sq Mean** | **Std Error** | **Mean** |
| --- | --- | --- | --- |
| 2015 | 26,222708 | 0,30367446 | 26,2227 |
| 2016 | 27,246566 | 0,25897445 | 27,2466 |
