# Supplementary material for: Inter- and intra-tree variability of carbon and oxygen stable isotope ratios of modern pollen from nine European tree species
Source: PLoS One. 2020 Jun 9;15(6):e0234315. doi: 10.1371/journal.pone.0234315 (PMC7282652; doi:10.1371/journal.pone.0234315)
Supplement: S2 Dataset — Nine variables influencing the stable isotope composition in pollen (δ13C, δ18O) at each site were explored by means of stepwise regression analysis. They include the continuous variable altitude and the categorical variables year (of sampling), month (of sampling), maturity (of the pollen at the time of sampling), slope, water (proximity to water body), water classification (type of water body), soil and species. (DOCX) [file pone.0234315.s002.docx]

**Detailed results of the Stepwise Regression Analysis: Modelling by Site**

1. **Parc Naturel Forêt d'Anlier**

**Environmental factors affecting δ^13^C**

**Response d13C**

**Whole Model**

**Actual by Predicted Plot**

**Effect Summary**

| **Source** | **LogWorth** |  | **PValue** |
| --- | --- | --- | --- |
| species{Alnus&Corylus-Fagus&Pinus&Quercus&Picea&Carpinus&Acer&Betula} | 28,179 |  | 0,00000 |
| species{Fagus&Pinus-Quercus&Picea&Carpinus&Acer&Betula} | 7,269 |  | 0,00000 |
| species{Quercus&Picea&Carpinus-Acer&Betula} | 3,412 |  | 0,00039 |
| year | 2,223 |  | 0,00599 |

**Lack Of Fit**

| **Source** | **DF** | **Sum of Squares** | **Mean Square** | **F Ratio** |
| --- | --- | --- | --- | --- |
| Lack Of Fit | 3 | 18,36594 | 6,12198 | 2,9744 |
| Pure Error | 254 | 522,79622 | 2,05825 | **Prob > F** |
| Total Error | 257 | 541,16216 |  | 0,0323* |
|  |  |  |  | **Max RSq** |
|  |  |  |  | 0,5295 |

**Residual by Predicted Plot**

**Summary of Fit**

| RSquare | 0,512999 |
| --- | --- |
| RSquare Adj | 0,50542 |
| Root Mean Square Error | 1,451099 |
| Mean of Response | -26,8952 |
| Observations (or Sum Wgts) | 262 |

**Analysis of Variance**

| **Source** | **DF** | **Sum of Squares** | **Mean Square** | **F Ratio** |
| --- | --- | --- | --- | --- |
| Model | 4 | 570,0523 | 142,513 | 67,6800 |
| Error | 257 | 541,1622 | 2,106 | **Prob > F** |
| C. Total | 261 | 1111,2145 |  | <,0001* |

**Parameter Estimates**

| **Term** | **Estimate** | **Std Error** | **t Ratio** | **Prob>\|t\|** | **VIF** |
| --- | --- | --- | --- | --- | --- |
| Intercept | -27,59724 | 0,10229 | -269,8 | <,0001* | . |
| species{Alnus&Corylus-Fagus&Pinus&Quercus&Picea&Carpinus&Acer&Betula} | -1,29421 | 0,10215 | -12,67 | <,0001* | 1,12402 |
| species{Fagus&Pinus-Quercus&Picea&Carpinus&Acer&Betula} | -0,7194 | 0,128368 | -5,60 | <,0001* | 1,1641955 |
| species{Quercus&Picea&Carpinus-Acer&Betula} | -0,464973 | 0,129291 | -3,60 | 0,0004* | 1,0432919 |
| year[2015] | -0,258387 | 0,093235 | -2,77 | 0,0060* | 1,0674249 |

**species{Alnus&Corylus-Fagus&Pinus&Quercus&Picea&Carpinus&Acer&Betula}**

**Leverage Plot**

**species{Fagus&Pinus-Quercus&Picea&Carpinus&Acer&Betula}**

**Leverage Plot**

**species{Quercus&Picea&Carpinus-Acer&Betula}**

**Leverage Plot**

**year**

**Leverage Plot**

**Least Squares Means Table**

| **Level** | **Least Sq Mean** | **Std Error** | **Mean** |
| --- | --- | --- | --- |
| 2015 | -27,18318 | 0,13723896 | -27,131 |
| 2016 | -26,66641 | 0,12187319 | -26,708 |

**Environmental factors affecting δ^18^O**

**Actual by Predicted Plot**

**Effect Summary**

| **Source** | **LogWorth** |  | **PValue** |
| --- | --- | --- | --- |
| month | 13,840 |  | 0,00000 |
| species{Alnus&Corylus&Fagus&Betula&Picea-Acer&Carpinus&Quercus&Pinus} | 12,143 |  | 0,00000 |
| species{Acer&Carpinus&Quercus-Pinus} | 6,258 |  | 0,00000 |
| species{Fagus-Betula} | 5,190 |  | 0,00001 |
| species{Fagus&Betula-Picea} | 3,686 |  | 0,00021 |
| year | 3,453 |  | 0,00035 |
| water_classification{2-1&0} | 2,871 |  | 0,00135 |
| maturity{-1-0} | 2,770 |  | 0,00170 |
| species{Acer-Carpinus&Quercus} | 2,377 |  | 0,00420 |
| species{Alnus&Corylus-Fagus&Betula&Picea} | 1,724 |  | 0,01886 |
| maturity{-1&0-1} | 0,258 |  | 0,55224 |

**Lack Of Fit**

| **Source** | **DF** | **Sum of Squares** | **Mean Square** | **F Ratio** |
| --- | --- | --- | --- | --- |
| Lack Of Fit | 19 | 95,36945 | 5,01944 | 5,0235 |
| Pure Error | 231 | 230,81261 | 0,99919 | **Prob > F** |
| Total Error | 250 | 326,18206 |  | <,0001* |
|  |  |  |  | **Max RSq** |
|  |  |  |  | 0,8416 |

**Residual by Predicted Plot**

**Studentized Residuals**

Externally studentized residuals with 95% simultaneous limits (Bonferroni) in red, individual limits in green.

**Parameter Estimates**

| **Term** | **Estimate** | **Std Error** | **t Ratio** | **Prob>\|t\|** | **VIF** |
| --- | --- | --- | --- | --- | --- |
| Intercept | 23,903648 | 0,244985 | 97,57 | <,0001* | . |
| species{Alnus&Corylus&Fagus&Betula&Picea-Acer&Carpinus&Quercus&Pinus} | -1,135806 | 0,150043 | -7,57 | <,0001* | 3,6946649 |
| species{Alnus&Corylus-Fagus&Betula&Picea} | 0,4976027 | 0,210526 | 2,36 | 0,0189* | 6,2951597 |
| species{Fagus&Betula-Picea} | -0,471946 | 0,125286 | -3,77 | 0,0002* | 1,204147 |
| species{Fagus-Betula} | -0,762262 | 0,165383 | -4,61 | <,0001* | 1,3627781 |
| species{Acer&Carpinus&Quercus-Pinus} | -0,912426 | 0,177493 | -5,14 | <,0001* | 1,7533328 |
| species{Acer-Carpinus&Quercus} | -0,726765 | 0,251533 | -2,89 | 0,0042* | 2,1573428 |
| year[2015] | -0,304774 | 0,08412 | -3,62 | 0,0004* | 1,4023376 |
| month[mar] | -1,693745 | 0,207071 | -8,18 | <,0001* | 7,9234161 |
| maturity{-1&0-1} | 0,1349925 | 0,226799 | 0,60 | 0,5522 | 1,2230564 |
| maturity{-1-0} | -0,460088 | 0,145 | -3,17 | 0,0017* | 1,3806857 |
| water_classification{2-1&0} | 0,348585 | 0,107502 | 3,24 | 0,0013* | 1,8174834 |

**Prediction Profiler**

1. **Parco Naturale Tre Cime**

**Environmental factors affecting δ^13^C**

**Response d13c**

**Whole Model**

**Actual by Predicted Plot**

**Effect Summary**

| **Source** | **LogWorth** |  | **PValue** |
| --- | --- | --- | --- |
| species{Fagus&Pinus&Acer-Betula&Picea} | 11,232 |  | 0,00000 |
| species{Betula-Picea} | 2,981 |  | 0,00104 |
| maturity{-1-0} | 2,071 |  | 0,00849 |
| maturity{-1&0-1} | 1,508 |  | 0,03108 |

**Lack Of Fit**

| **Source** | **DF** | **Sum of Squares** | **Mean Square** | **F Ratio** |
| --- | --- | --- | --- | --- |
| Lack Of Fit | 3 | 2,923279 | 0,974426 | 1,1348 |
| Pure Error | 62 | 53,236913 | 0,858660 | **Prob > F** |
| Total Error | 65 | 56,160192 |  | 0,3420 |
|  |  |  |  | **Max RSq** |
|  |  |  |  | 0,7472 |

**Residual by Predicted Plot**

**Summary of Fit**

| RSquare | 0,73335 |
| --- | --- |
| RSquare Adj | 0,716941 |
| Root Mean Square Error | 0,929518 |
| Mean of Response | -25,4139 |
| Observations (or Sum Wgts) | 70 |

**Analysis of Variance**

| **Source** | **DF** | **Sum of Squares** | **Mean Square** | **F Ratio** |
| --- | --- | --- | --- | --- |
| Model | 4 | 154,45401 | 38,6135 | 44,6914 |
| Error | 65 | 56,16019 | 0,8640 | **Prob > F** |
| C. Total | 69 | 210,61420 |  | <,0001* |

**Parameter Estimates**

| **Term** | **Estimate** | **Std Error** | **t Ratio** | **Prob>\|t\|** | **VIF** |
| --- | --- | --- | --- | --- | --- |
| Intercept | -25,65596 | 0,172831 | -148,4 | <,0001* | . |
| species{Fagus&Pinus&Acer-Betula&Picea} | -1,273705 | 0,151766 | -8,39 | <,0001* | 1,7685937 |
| species{Betula-Picea} | -0,883425 | 0,25736 | -3,43 | 0,0010* | 1,6744641 |
| maturity{-1&0-1} | 0,3389937 | 0,153814 | 2,20 | 0,0311* | 1,2908939 |
| maturity{-1-0} | -0,36713 | 0,135236 | -2,71 | 0,0085* | 1,0550521 |

**species{Fagus&Pinus&Acer-Betula&Picea}**

**Leverage Plot**

**species{Betula-Picea}**

**Leverage Plot**

**maturity{-1&0-1}**

**Leverage Plot**

**maturity{-1-0}**

**Leverage Plot**

**Environmental factors affecting δ^18^O**

**Response d18o**

**Whole Model**

**Regression Plot**

**Actual by Predicted Plot**

**Effect Summary**

| **Source** | **LogWorth** |  | **PValue** |
| --- | --- | --- | --- |
| year | 9,013 |  | 0,00000 |
| species{Picea-Acer&Betula&Fagus&Pinus} | 8,668 |  | 0,00000 |

**Lack Of Fit**

| **Source** | **DF** | **Sum of Squares** | **Mean Square** | **F Ratio** |
| --- | --- | --- | --- | --- |
| Lack Of Fit | 1 | 0,27594 | 0,27594 | 0,1780 |
| Pure Error | 66 | 102,33003 | 1,55045 | **Prob > F** |
| Total Error | 67 | 102,60597 |  | 0,6745 |
|  |  |  |  | **Max RSq** |
|  |  |  |  | 0,6189 |

**Residual by Predicted Plot**

**Summary of Fit**

| RSquare | 0,617917 |
| --- | --- |
| RSquare Adj | 0,606511 |
| Root Mean Square Error | 1,237511 |
| Mean of Response | 23,94726 |
| Observations (or Sum Wgts) | 70 |

**Analysis of Variance**

| **Source** | **DF** | **Sum of Squares** | **Mean Square** | **F Ratio** |
| --- | --- | --- | --- | --- |
| Model | 2 | 165,93758 | 82,9688 | 54,1772 |
| Error | 67 | 102,60597 | 1,5314 | **Prob > F** |
| C. Total | 69 | 268,54355 |  | <,0001* |

**Parameter Estimates**

| **Term** | **Estimate** | **Std Error** | **t Ratio** | **Prob>\|t\|** | **VIF** |
| --- | --- | --- | --- | --- | --- |
| Intercept | 23,663193 | 0,158457 | 149,34 | <,0001* | . |
| species{Picea-Acer&Betula&Fagus&Pinus} | -1,093478 | 0,158132 | -6,91 | <,0001* | 1,0086215 |
| year[2015] | 1,059764 | 0,149096 | 7,11 | <,0001* | 1,0086215 |

**species{Picea-Acer&Betula&Fagus&Pinus}**

**Leverage Plot**

**year**

**Leverage Plot**

**Least Squares Means Table**

| **Level** | **Least Sq Mean** | **Std Error** | **Mean** |
| --- | --- | --- | --- |
| 2015 | 25,097864 | 0,21927436 | 25,2014 |
| 2016 | 22,978336 | 0,20114594 | 22,8912 |

1. **Liesjärvi National Park**

**Environmental factors affecting δ^13^C**

**Response d13c**

**Whole Model**

**Actual by Predicted Plot**

**Residual by Predicted Plot**

**Summary of Fit**

| RSquare | 0,674006 |
| --- | --- |
| RSquare Adj | 0,662363 |
| Root Mean Square Error | 0,806806 |
| Mean of Response | -27,132 |
| Observations (or Sum Wgts) | 30 |

**Analysis of Variance**

| **Source** | **DF** | **Sum of Squares** | **Mean Square** | **F Ratio** |
| --- | --- | --- | --- | --- |
| Model | 1 | 37,683375 | 37,6834 | 57,8911 |
| Error | 28 | 18,226194 | 0,6509 | **Prob > F** |
| C. Total | 29 | 55,909569 |  | <,0001* |

**Parameter Estimates**

| **Term** | **Estimate** | **Std Error** | **t Ratio** | **Prob>\|t\|** | **VIF** |
| --- | --- | --- | --- | --- | --- |
| Intercept | -26,73575 | 0,156237 | -171,1 | <,0001* | . |
| species[Picea] | 1,18875 | 0,156237 | 7,61 | <,0001* | 1 |

**species**

**Leverage Plot**

**Least Squares Means Table**

| **Level** | **Least Sq Mean** | **Std Error** | **Mean** |
| --- | --- | --- | --- |
| Picea | -25,54700 | 0,25513438 | -25,547 |
| Pinus | -27,92450 | 0,18040725 | -27,925 |

**Environmental factors affecting δ^18^O**

**Response d18o**

**Whole Model**

**Actual by Predicted Plot**

**Residual by Predicted Plot**

**Summary of Fit**

| RSquare | 0,276816 |
| --- | --- |
| RSquare Adj | 0,250988 |
| Root Mean Square Error | 0,719807 |
| Mean of Response | 23,28578 |
| Observations (or Sum Wgts) | 30 |

**Analysis of Variance**

| **Source** | **DF** | **Sum of Squares** | **Mean Square** | **F Ratio** |
| --- | --- | --- | --- | --- |
| Model | 1 | 5,553070 | 5,55307 | 10,7177 |
| Error | 28 | 14,507418 | 0,51812 | **Prob > F** |
| C. Total | 29 | 20,060487 |  | 0,0028* |

**Parameter Estimates**

| **Term** | **Estimate** | **Std Error** | **t Ratio** | **Prob>\|t\|** | **VIF** |
| --- | --- | --- | --- | --- | --- |
| Intercept | 23,133667 | 0,13939 | 165,96 | <,0001* | . |
| species[Picea] | -0,456333 | 0,13939 | -3,27 | 0,0028* | 1 |

**species**

**Leverage Plot**

**Least Squares Means Table**

| **Level** | **Least Sq Mean** | **Std Error** | **Mean** |
| --- | --- | --- | --- |
| Picea | 22,677333 | 0,22762295 | 22,6773 |
| Pinus | 23,590000 | 0,16095373 | 23,5900 |

1. **Gorczański Park Narodowy**

**Environmental factors affecting δ^13^C**

**Response d13c**

**Whole Model**

**Actual by Predicted Plot**

**Effect Summary**

| **Source** | **LogWorth** |  | **PValue** |
| --- | --- | --- | --- |
| species{Fagus&Corylus&Alnus&Pinus-Picea&Acer} | 10,721 |  | 0,00000 |
| soil1 | 3,205 |  | 0,00062 |
| year | 2,019 |  | 0,00958 |
| water{2-0&1} | 1,754 |  | 0,01760 |

**Lack Of Fit**

| **Source** | **DF** | **Sum of Squares** | **Mean Square** | **F Ratio** |
| --- | --- | --- | --- | --- |
| Lack Of Fit | 5 | 16,35214 | 3,27043 | 1,7837 |
| Pure Error | 187 | 342,86307 | 1,83349 | **Prob > F** |
| Total Error | 192 | 359,21521 |  | 0,1181 |
|  |  |  |  | **Max RSq** |
|  |  |  |  | 0,3021 |

**Residual by Predicted Plot**

**Summary of Fit**

| RSquare | 0,268764 |
| --- | --- |
| RSquare Adj | 0,25353 |
| Root Mean Square Error | 1,367813 |
| Mean of Response | -26,6005 |
| Observations (or Sum Wgts) | 197 |

**Analysis of Variance**

| **Source** | **DF** | **Sum of Squares** | **Mean Square** | **F Ratio** |
| --- | --- | --- | --- | --- |
| Model | 4 | 132,02898 | 33,0072 | 17,6423 |
| Error | 192 | 359,21521 | 1,8709 | **Prob > F** |
| C. Total | 196 | 491,24419 |  | <,0001* |

**Parameter Estimates**

| **Term** | **Estimate** | **Std Error** | **t Ratio** | **Prob>\|t\|** | **VIF** |
| --- | --- | --- | --- | --- | --- |
| Intercept | -26,26759 | 0,243982 | -107,7 | <,0001* | . |
| species{Fagus&Corylus&Alnus&Pinus-Picea&Acer} | -0,839238 | 0,117578 | -7,14 | <,0001* | 1,0880601 |
| year[2015] | -0,260736 | 0,099634 | -2,62 | 0,0096* | 1,0391985 |
| water{2-0&1} | -0,475893 | 0,198735 | -2,39 | 0,0176* | 1,0252988 |
| soil1[EF] | 0,472104 | 0,135729 | 3,48 | 0,0006* | 1,1336311 |

**species{Fagus&Corylus&Alnus&Pinus-Picea&Acer}**

**Leverage Plot**

**year**

**Leverage Plot**

**Least Squares Means Table**

| **Level** | **Least Sq Mean** | **Std Error** | **Mean** |
| --- | --- | --- | --- |
| 2015 | -26,53699 | 0,15117571 | -26,822 |
| 2016 | -26,01552 | 0,17854524 | -26,343 |

**water{2-0&1}**

**Leverage Plot**

**soil1**

**Leverage Plot**

**Least Squares Means Table**

| **Level** | **Least Sq Mean** | **Std Error** | **Mean** |
| --- | --- | --- | --- |
| EF | -25,80415 | 0,24488599 | -26,285 |
| HL-DC | -26,74836 | 0,10841891 | -26,669 |

**Environmental factors affecting δ^18^O**

**Response d18o**

**Whole Model**

**Actual by Predicted Plot**

**Effect Summary**

| **Source** | **LogWorth** |  | **PValue** |
| --- | --- | --- | --- |
| species{Acer&Alnus&Corylus&Fagus-Picea&Pinus} | 47,708 |  | 0,00000 |
| species{Picea-Pinus} | 4,311 |  | 0,00005 |
| month{may-june} | 2,670 |  | 0,00214 |
| Altitude | 1,691 |  | 0,02037 |
| month{march-may&june} | 0,782 |  | 0,16515 |

**Lack Of Fit**

| **Source** | **DF** | **Sum of Squares** | **Mean Square** | **F Ratio** |
| --- | --- | --- | --- | --- |
| Lack Of Fit | 88 | 207,82287 | 2,36162 | 2,3186 |
| Pure Error | 103 | 104,91215 | 1,01856 | **Prob > F** |
| Total Error | 191 | 312,73502 |  | <,0001* |
|  |  |  |  | **Max RSq** |
|  |  |  |  | 0,9509 |

**Residual by Predicted Plot**

**Summary of Fit**

| RSquare | 0,853646 |
| --- | --- |
| RSquare Adj | 0,849814 |
| Root Mean Square Error | 1,279592 |
| Mean of Response | 20,913 |
| Observations (or Sum Wgts) | 197 |

**Analysis of Variance**

| **Source** | **DF** | **Sum of Squares** | **Mean Square** | **F Ratio** |
| --- | --- | --- | --- | --- |
| Model | 5 | 1824,0991 | 364,820 | 222,8103 |
| Error | 191 | 312,7350 | 1,637 | **Prob > F** |
| C. Total | 196 | 2136,8341 |  | <,0001* |

**Parameter Estimates**

| **Term** | **Estimate** | **Std Error** | **t Ratio** | **Prob>\|t\|** | **VIF** |
| --- | --- | --- | --- | --- | --- |
| Intercept | 25,035346 | 1,374577 | 18,21 | <,0001* | . |
| species{Acer&Alnus&Corylus&Fagus-Picea&Pinus} | -2,705796 | 0,136003 | -19,90 | <,0001* | 2,0643892 |
| species{Picea-Pinus} | -0,838595 | 0,201797 | -4,16 | <,0001* | 1,7826038 |
| Altitude | -0,00458 | 0,001958 | -2,34 | 0,0204* | 2,3001503 |
| month{march-may&june} | -0,239863 | 0,172154 | -1,39 | 0,1651 | 3,4988143 |
| month{may-june} | -0,640193 | 0,205675 | -3,11 | 0,0021* | 2,0957115 |

**species{Acer&Alnus&Corylus&Fagus-Picea&Pinus}**

**Leverage Plot**

**species{Picea-Pinus}**

**Leverage Plot**

**Altitude**

**Leverage Plot**

**month{march-may&june}**

**Leverage Plot**

**month{may-june}**

**Leverage Plot**

1. **Müritz Nationalpark**

**Environmental factors affecting δ^13^C**

**Response d13c**

**Whole Model**

**Actual by Predicted Plot**

**Effect Summary**

| **Source** | **LogWorth** |  | **PValue** |
| --- | --- | --- | --- |
| species{Alnus-Pinus&Corylus&Betula} | 5,729 |  | 0,00000 |
| species{Pinus-Corylus&Betula} | 1,235 |  | 0,05820 |
| month{feb&may-apr} | 1,168 |  | 0,06788 |

**Residual by Predicted Plot**

**Summary of Fit**

| RSquare | 0,787241 |
| --- | --- |
| RSquare Adj | 0,759489 |
| Root Mean Square Error | 1,438416 |
| Mean of Response | -26,1694 |
| Observations (or Sum Wgts) | 27 |

**Analysis of Variance**

| **Source** | **DF** | **Sum of Squares** | **Mean Square** | **F Ratio** |
| --- | --- | --- | --- | --- |
| Model | 3 | 176,08216 | 58,6941 | 28,3678 |
| Error | 23 | 47,58793 | 2,0690 | **Prob > F** |
| C. Total | 26 | 223,67010 |  | <,0001* |

**Parameter Estimates**

| **Term** | **Estimate** | **Std Error** | **t Ratio** | **Prob>\|t\|** | **VIF** |
| --- | --- | --- | --- | --- | --- |
| Intercept | -27,81631 | 0,44529 | -62,47 | <,0001* | . |
| species{Alnus-Pinus&Corylus&Betula} | -2,421778 | 0,382828 | -6,33 | <,0001* | 1,154321 |
| species{Pinus-Corylus&Betula} | -0,827778 | 0,415235 | -1,99 | 0,0582 | 1,5246914 |
| month{feb&may-apr} | -0,711583 | 0,371397 | -1,92 | 0,0679 | 1,6790123 |

**species{Alnus-Pinus&Corylus&Betula}**

**Leverage Plot**

**species{Pinus-Corylus&Betula}**

**Leverage Plot**

**month{feb&may-apr}**

**Leverage Plot**

**Environmental factors affecting δ^18^O**

**Response d18o**

**Whole Model**

**Actual by Predicted Plot**

**Effect Summary**

| **Source** | **LogWorth** |  | **PValue** |
| --- | --- | --- | --- |
| species{Alnus&Corylus&Betula-Pinus} | 17,633 |  | 0,00000 |
| species{Alnus-Corylus&Betula} | 5,822 |  | 0,00000 |

**Residual by Predicted Plot**

**Summary of Fit**

| RSquare | 0,960571 |
| --- | --- |
| RSquare Adj | 0,957286 |
| Root Mean Square Error | 0,472313 |
| Mean of Response | 24,77831 |
| Observations (or Sum Wgts) | 27 |

**Analysis of Variance**

| **Source** | **DF** | **Sum of Squares** | **Mean Square** | **F Ratio** |
| --- | --- | --- | --- | --- |
| Model | 2 | 130,43341 | 65,2167 | 292,3469 |
| Error | 24 | 5,35392 | 0,2231 | **Prob > F** |
| C. Total | 26 | 135,78732 |  | <,0001* |

**Parameter Estimates**

| **Term** | **Estimate** | **Std Error** | **t Ratio** | **Prob>\|t\|** | **VIF** |
| --- | --- | --- | --- | --- | --- |
| Intercept | 25,994861 | 0,11382 | 228,39 | <,0001* | . |
| species{Alnus&Corylus&Betula-Pinus} | -2,751806 | 0,11382 | -24,18 | <,0001* | 1,0840278 |
| species{Alnus-Corylus&Betula} | -0,766389 | 0,120994 | -6,33 | <,0001* | 1,0840278 |

**species{Alnus&Corylus&Betula-Pinus}**

**Leverage Plot**

**species{Alnus-Corylus&Betula}**

**Leverage Plot**

1. **Steigerwald Nationalpark**

**Environmental factors affecting δ^13^C**

**Response d13c**

**Whole Model**

**Regression Plot**

**Actual by Predicted Plot**

**Residual by Predicted Plot**

**Summary of Fit**

| RSquare | 0,48827 |
| --- | --- |
| RSquare Adj | 0,478794 |
| Root Mean Square Error | 1,25566 |
| Mean of Response | -25,1117 |
| Observations (or Sum Wgts) | 56 |

**Analysis of Variance**

| **Source** | **DF** | **Sum of Squares** | **Mean Square** | **F Ratio** |
| --- | --- | --- | --- | --- |
| Model | 1 | 81,23777 | 81,2378 | 51,5245 |
| Error | 54 | 85,14088 | 1,5767 | **Prob > F** |
| C. Total | 55 | 166,37865 |  | <,0001* |

**Parameter Estimates**

| **Term** | **Estimate** | **Std Error** | **t Ratio** | **Prob>\|t\|** | **VIF** |
| --- | --- | --- | --- | --- | --- |
| Intercept | -24,48031 | 0,189452 | -129,2 | <,0001* | . |
| species{Corylus&Alnus&Pinus&Picea&Carpinus&Fagus-Acer&Quercus&Betula} | -1,359895 | 0,189452 | -7,18 | <,0001* | 1 |

**species{Corylus&Alnus&Pinus&Picea&Carpinus&Fagus-Acer&Quercus&Betula}**

**Leverage Plot**

**Environmental factors affecting δ^18^O**

**Response d18o**

**Whole Model**

**Actual by Predicted Plot**

**Effect Summary**

| **Source** | **LogWorth** |  | **PValue** |
| --- | --- | --- | --- |
| year | 7,949 |  | 0,00000 |
| species{Alnus&Fagus&Corylus&Picea-Acer&Betula} | 6,155 |  | 0,00000 |
| species{Alnus&Fagus&Corylus&Picea&Acer&Betula-Carpinus&Quercus&Pinus} | 2,760 |  | 0,00174 |
| Altitude | 2,560 |  | 0,00276 |

**Lack Of Fit**

| **Source** | **DF** | **Sum of Squares** | **Mean Square** | **F Ratio** |
| --- | --- | --- | --- | --- |
| Lack Of Fit | 27 | 35,212659 | 1,30417 | 2,7186 |
| Pure Error | 24 | 11,513320 | 0,47972 | **Prob > F** |
| Total Error | 51 | 46,725980 |  | 0,0078* |
|  |  |  |  | **Max RSq** |
|  |  |  |  | 0,9373 |

**Residual by Predicted Plot**

**Summary of Fit**

| RSquare | 0,745426 |
| --- | --- |
| RSquare Adj | 0,72546 |
| Root Mean Square Error | 0,957181 |
| Mean of Response | 24,75588 |
| Observations (or Sum Wgts) | 56 |

**Analysis of Variance**

| **Source** | **DF** | **Sum of Squares** | **Mean Square** | **F Ratio** |
| --- | --- | --- | --- | --- |
| Model | 4 | 136,81987 | 34,2050 | 37,3337 |
| Error | 51 | 46,72598 | 0,9162 | **Prob > F** |
| C. Total | 55 | 183,54585 |  | <,0001* |

**Parameter Estimates**

| **Term** | **Estimate** | **Std Error** | **t Ratio** | **Prob>\|t\|** | **VIF** |
| --- | --- | --- | --- | --- | --- |
| Intercept | 20,898251 | 1,600358 | 13,06 | <,0001* | . |
| species{Alnus&Fagus&Corylus&Picea&Acer&Betula-Carpinus&Quercus&Pinus} | -0,640149 | 0,193636 | -3,31 | 0,0017* | 1,9994661 |
| species{Alnus&Fagus&Corylus&Picea-Acer&Betula} | -1,026962 | 0,181488 | -5,66 | <,0001* | 1,2403039 |
| year[2015] | 1,3052398 | 0,191927 | 6,80 | <,0001* | 1,5163089 |
| Altitude | 0,0130831 | 0,004158 | 3,15 | 0,0028* | 2,0941782 |

**species{Alnus&Fagus&Corylus&Picea&Acer&Betula-Carpinus&Quercus&Pinus}**

**Leverage Plot**

**species{Alnus&Fagus&Corylus&Picea-Acer&Betula}**

**Leverage Plot**

**year**

**Leverage Plot**

**Least Squares Means Table**

| **Level** | **Least Sq Mean** | **Std Error** | **Mean** |
| --- | --- | --- | --- |
| 2015 | 26,806975 | 0,32760113 | 26,2153 |
| 2016 | 24,196496 | 0,15207365 | 24,3579 |

**Altitude**

**Leverage Plot**

1. **Tatrzański Park Narodowy**

**Environmental factors affecting δ^13^C**

**Response d13c**

**Whole Model**

**Actual by Predicted Plot**

**Effect Summary**

| **Source** | **LogWorth** |  | **PValue** |
| --- | --- | --- | --- |
| Altitude | 2,586 |  | 0,00259 |
| maturity | 1,332 |  | 0,04652 |
| year | 1,041 |  | 0,09106 |

**Lack Of Fit**

| **Source** | **DF** | **Sum of Squares** | **Mean Square** | **F Ratio** |
| --- | --- | --- | --- | --- |
| Lack Of Fit | 3 | 0,3163198 | 0,105440 | 0,1371 |
| Pure Error | 9 | 6,9203373 | 0,768926 | **Prob > F** |
| Total Error | 12 | 7,2366571 |  | 0,9353 |
|  |  |  |  | **Max RSq** |
|  |  |  |  | 0,7383 |

**Residual by Predicted Plot**

**Summary of Fit**

| RSquare | 0,726307 |
| --- | --- |
| RSquare Adj | 0,635076 |
| Root Mean Square Error | 0,776566 |
| Mean of Response | -24,6134 |
| Observations (or Sum Wgts) | 17 |

**Analysis of Variance**

| **Source** | **DF** | **Sum of Squares** | **Mean Square** | **F Ratio** |
| --- | --- | --- | --- | --- |
| Model | 4 | 19,204090 | 4,80102 | 7,9612 |
| Error | 12 | 7,236657 | 0,60305 | **Prob > F** |
| C. Total | 16 | 26,440747 |  | 0,0023* |

**Parameter Estimates**

| **Term** | **Estimate** | **Std Error** | **t Ratio** | **Prob>\|t\|** | **VIF** |
| --- | --- | --- | --- | --- | --- |
| Intercept | -36,884 | 3,081296 | -11,97 | <,0001* | . |
| year[2015] | 0,5327188 | 0,289964 | 1,84 | 0,0911 | 1,3778189 |
| Altitude | 0,0106151 | 0,002803 | 3,79 | 0,0026* | 2,9192483 |
| maturity[-1] | -1,484498 | 0,551652 | -2,69 | 0,0196* | 2,4934729 |
| maturity[0] | -0,296114 | 0,302738 | -0,98 | 0,3473 | 1,2336918 |

**Effect Tests**

| **Source** | **Nparm** | **DF** | **Sum of Squares** | **F Ratio** | **Prob > F** |
| --- | --- | --- | --- | --- | --- |
| year | 1 | 1 | 2,0354680 | 3,3753 | 0,0911 |
| Altitude | 1 | 1 | 8,6471003 | 14,3388 | 0,0026* |
| maturity | 2 | 2 | 4,8304143 | 4,0050 | 0,0465* |

**year**

**Leverage Plot**

**Least Squares Means Table**

| **Level** | **Least Sq Mean** | **Std Error** | **Mean** |
| --- | --- | --- | --- |
| 2015 | -24,16390 | 0,25271449 | -24,249 |
| 2016 | -25,22934 | 0,60672045 | -26,312 |

**Altitude**

**Leverage Plot**

**maturity**

**Leverage Plot**

**Least Squares Means Table**

| **Level** | **Least Sq Mean** | **Std Error** | **Mean** |
| --- | --- | --- | --- |
| -1 | -26,18112 | 0,58041203 | -24,160 |
| 0 | -24,99274 | 0,27009594 | -24,771 |
| 1 | -22,91601 | 0,88910917 | -24,348 |

**Environmental factors affecting δ^18^O**

**Response d18o**

**Whole Model**

**Actual by Predicted Plot**

**Residual by Predicted Plot**

**Summary of Fit**

| RSquare | 0,347604 |
| --- | --- |
| RSquare Adj | 0,304111 |
| Root Mean Square Error | 0,729891 |
| Mean of Response | 22,36963 |
| Observations (or Sum Wgts) | 17 |

**Analysis of Variance**

| **Source** | **DF** | **Sum of Squares** | **Mean Square** | **F Ratio** |
| --- | --- | --- | --- | --- |
| Model | 1 | 4,257751 | 4,25775 | 7,9922 |
| Error | 15 | 7,991111 | 0,53274 | **Prob > F** |
| C. Total | 16 | 12,248862 |  | 0,0127* |

**Parameter Estimates**

| **Term** | **Estimate** | **Std Error** | **t Ratio** | **Prob>\|t\|** | **VIF** |
| --- | --- | --- | --- | --- | --- |
| Intercept | 21,944906 | 0,232182 | 94,52 | <,0001* | . |
| year[2015] | 0,656387 | 0,232182 | 2,83 | 0,0127* | 1 |

**year**

**Leverage Plot**

**Least Squares Means Table**

| **Level** | **Least Sq Mean** | **Std Error** | **Mean** |
| --- | --- | --- | --- |
| 2015 | 22,601293 | 0,19507155 | 22,6013 |
| 2016 | 21,288519 | 0,42140270 | 21,2885 |
